# Checklist of tapeworms (Platyhelminthes, Cestoda) of vertebrates in Finland

**DOI:** 10.3897/zookeys.533.6538

**Published:** 2015-11-09

**Authors:** Voitto Haukisalmi

**Affiliations:** 1 Finnish Museum of Natural History Luomus, P. O. Box 17, P. Rautatiekatu 13, 00014 University of Helsinki, Finland

**Keywords:** Cestoda, tapeworms, fishes, birds, mammals, checklist, fauna, Finland, species diversity

## Abstract

A checklist of tapeworms (Cestoda) of vertebrates (fishes, birds and mammals) in Finland is presented, based on published observations, specimens deposited in the collections of the Finnish Museum of Natural History (Helsinki) and the Zoological Museum of the University of Turku, and additional specimens identified by the present author. The checklist includes 170 tapeworm species from 151 host species, comprising 447 parasite species/host species combinations. Thirty of the tapeworm species and 96 of the parasite/host species combinations have not been previously reported from Finland. The total number of tapeworm species in Finland (170 spp.) is significantly lower than the corresponding figure for the Iberian Peninsula (257 spp.), Slovakia (225 spp.) and Poland (279 spp.). The difference between Finland and the other three regions is particularly pronounced for anseriform, podicipediform, charadriiform and passeriform birds, reflecting inadequate and/or biased sampling of these birds in Finland. It is predicted that there are actually ca. 270 species of tapeworms in Finland, assuming that true number of bird tapeworms in Finland corresponds to that in other European countries with more comprehensive knowledge of the local tapeworm fauna. The other main pattern emerging from the present data is the seemingly unexplained absence in (northern) Fennoscandia of several mammalian tapeworms that otherwise have extensive distributions in the Holarctic region or in Eurasia, including the northern regions. Previously unknown type specimens, that is, the holotype of *Bothrimonus nylandicus* Schneider, 1902 (a junior synonym of *Diplocotyle olrikii* Krabbe, 1874) (MZH 127096) and the syntypes of *Caryophyllaeides fennica* (Schneider, 1902) (MZH 127097) were located in the collections of the Finnish Museum of Natural History.

## Introduction

There are no comprehensive checklists or other faunistic reviews of tapeworms (Cestoda) of vertebrates in northern Europe, although the cestodes of fishes have been recently reviewed in Latvia (Kirjušina and Vismanis 2007) and Finland (Pulkkinen and Valtonen 2012). Among other host groups, the cestode fauna of rodents and shrews has been intensively studied in northern Europe (see, for example, Haukisalmi 1986, 1989, Haukisalmi et al. 1994, Bugmyrin et al. 2003, Anikanova et al. 2007). However, the cestode fauna of birds and large mammals in northern Europe has received surpirisingly little attention, with the exception of a recent series of studies on taeniid cestodes of carnivores in Finland and Sweden (Lavikainen et al. 2006, 2011, 2013, Haukisalmi et al. 2011).

Comprehensive checklists of cestodes covering all vertebrate groups have, however, been published at least for France (Joyeux and Baer 1936), Spain and Portugal (Cordero del Campillo et al. 1994), Slovakia (Synopsis of cestodes in Slovakia I–V: Macko et al. 1993, 1994, Hanzelová et al. 1995, Hanzelová and Ryšavý 1996, 1999), Poland (Pojmańska et al. 2007) and Belarus (Merkusheva and Bobkova 1981). Because of recent developments in tapeworm taxonomy, the older checklists, such as those of Joyeux and Baer (1936), are naturally somewhat outdated. Tapeworm taxonomy has long flourished in Russia and the former USSR, resulting in major faunistical and systematical reviews of cestodes of all vertebrate groups. The most appropriate example is the “Essentials (or Fundamentals) of Cestodology” – series, started in 1951, and now including 14 volumes. However, there are evidently no proper checklists or faunistic reviews summarizing information on tapeworms of all vertebrate classes in the European part of Russia.

The main purpose of the present study is to provide a comprehensive list of tapeworm species reported or found from Finland, including two of the former Finnish territories lost as a consequence of the Second World War (Karelia and Petsamo regions). The study concerns all vertebrate groups present in Finland, but no tapeworms are known from Finnish elasmobranchs, amphibians and reptiles. Besides published reports, specimens deposited in the collections of the two major Finnish natural history museums were examined for the presence of otherwise unknown species. The present checklist also includes as yet undescribed, more or less cryptic mammalian tapeworms identified by molecular methods (for example, Haukisalmi et al. 2008, 2009a, Lavikainen et al. 2013).

The present faunistic data from Finland are compared with the existing checklists from Europe, particularly the most recent ones from the Iberian Peninsula, Slovakia and Poland. These comparisons allow the identification of host and cestode groups that need to be examined more comprehensively to obtain a better idea of the overall cestode diversity in Finland and northern Europe in general.

## Materials and methods

The list of tapeworm species of Finland, including the former territories in northern and south-eastern parts of the country, is based on published observations, specimens deposited in the collections of the Finnish Museum of Natural History, Helsinki (MZH) and the Zoological Museum of the University of Turku, Finland (ZMUT), as well as additional specimens identified by the present author. For each cestode species, all known definitive and intermediate host species are listed with references for published records. The checklist does not, however, provide a complete list of references. Instead, the first known reference and, if available, one or more recent ones with additional information on the particular cestode species, such as DNA sequence data, distribution and biology, is given for each cestode species/host species combination. The checklist does not include regions or localities for the cestode records, except for the former Finnish territories.

When specimens of a particular cestode species have been deposited in museum collections (in Finland or elsewhere), this has been indicated in the list, separately for each host species. However, collection/accession numbers are still unavailable for most of the specimens deposited in the Finnish museums (Helsinki and Turku). The specimens in the collections of both Finnish museums are generally old, commonly from the early 20^th^ century. Most of the specimens in the Finnish Museum of Natural History are stored in 80% ethanol (originally usually in formaldehyde), whereas the entire material in the Turku museum consists of specimens on slides.

Most of the cestodes are reported in their hosts are the adult stages, mainly because the metacestodes of most tapeworms parasitize invertebrates, which were excluded from the present list. Also, there is limited information on metacestodes parasitizing invertebrates from Finland, most of the existing data coming from the parasites of fishes (Valtonen et al. 2012). *Diphyllobothrium dendriticum* (Nitzsch, 1824), *Schistocephalus cotti* Chubb, Seppälä, Lüscher, Milinski & Valtonen, 2006, *Schistocephalus pungitii* Dubinina, 1959, *Taenia martis* (Zeder, 1803), *Versteria mustelae* (Gmelin, 1790), *Echinococcus equinus* Williams & Sweatman, 1963 and *Echinococcus granulosus* (Batsch, 1786) are only known as metacestodes from Finland.

Three workers stand out as collectors of older museum specimens of Finnish cestodes. Kaarlo M. Levander (1867–1943) and Guido Schneider (1867–1948) collected cestodes and other helminths of marine and freshwater fishes from Finland. The latter also published several faunistic and taxonomic papers on fish tapeworms, including descriptions of new taxa (e.g. Schneider 1902b, 1904, 1905). Knowledge of the tapeworm fauna of Finnish birds is based largely on the collections and original identifications of Väinö H. Pekkola (1880–1953). Pekkola never published any data on tapeworms he collected, but fortunately a major part of his extensive collections is deposited in MZH and ZMUT.

Tapeworms available for study (other than museum specimens) originate from three main sources. Practically all the existing knowledge of the Finnish tapeworm fauna of rodents and shrews is based on specimens collected in connection with research projects led by Heikki Henttonen (Natural Resources Institute Finland Luke, previously Finnish Forest Research Institute) from the late 1970’s until the present. Several tapeworm species and tapeworm/host species combinations new to Finland were identified among the tapeworms collected by specialists at the Finnish Safety Authority Evira (Marja Isomursu, Antti Oksanen). In addition, Antti Lavikainen (Haartman Institute, University of Helsinki) has recently collected and identified (by molecular methods) several taeniid species and taeniid/host species combinations new to Finland.

The geographical distribution of tapeworms of the field vole *Microtus agrestis* in Fennoscandia (Fig. 2) is based partly on published sources (Haukisalmi 1986, Haukisalmi et al. 1994, 2004, 2009a) and partly on the tapeworm collections of H. Henttonen, V. Haukisalmi and coworkers from Finland, northern Norway and Denmark, and on the field vole material collected by Maarit Jaarola from Sweden (Jaarola and Tegelström 1995, 1996, Jaarola et al. 1997).

The identifications of vouchers and other specimens deposited in museum collections were checked, except when the specimens were in poor condition or when the rostellar hooks were lacking. The original identifications of cestodes without existing voucher specimens were accepted as such, the names modified to follow current taxonomy. The latter was derived from several sources, the seminal book “Keys to the cestode parasites of vertebrates” (Khalil et al. 1994) forming the backbone of the genus-level classification. However, the genus name *Passerilepis* Spasskii & Spasskaya, 1954 has been used for *Microsomacanthus* Lopez-Neyra, 1942 –like cestodes parasitizing passerine birds, instead of merging them with the latter genus. Other major deviations from the classification scheme of Khalil et al. (1994) concern the *Anoplocephaloides* Baer, 1923 and *Paranoplocephala* Lühe, 1910 -like species (Anoplocephalidae) of rodents and *Taenia* Linnaeus, 1758 -like species (Taeniidae) of carnivores, recently revised by Haukisalmi (2009) and Haukisalmi et al. (2014), and Nakao et al. (2013), respectively.

Species-level taxonomy and identification are based on publications too numerous to be listed here, but the following books and papers may be mentioned as particularly important sources: Joyeux and Baer 1936 (all tapeworms), Scholz et al. 2007 (*Proteocephalus*), Spasskaya 1966 (hymenolepidids of birds), Spasskaya and Spasskii 1977, 1978 (dilepidids of birds), Matevosyan 1969 (paruterinids of birds), Spasskii 1951, Rausch 1976, Beveridge 1978 (anoplocephalids), Vaucher 1971 (tapeworms of shrews) and Abuladze 1964 (taeniids). However, recent changes in species names have also been considered.

Tapeworms that could not be identified to species were included in the list if they were morphologically clearly different from other (congeneric) species. The checklist includes only those synonyms and misidentifications that have been used in publications concerning the Finnish cestode fauna or in museum specimens.

The scientific names of hosts follow Froese and Pauly (2015, fishes), Dickinson and Remsen (2013, birds), Dickinson and Christidis (2014, birds) and Wilson and Reeder (2005, mammals).

## Results

The present checklist of tapeworms of Finland includes 170 parasite species from 151 host species, comprising 447 parasite species/host species combinations (see Appendix). Fishes, birds and mammals have 31, 80 and 67 tapeworm species, respectively. There is a slight overlap in the tapeworm faunas of the three main host groups, because the life-cycles of diphyllobothriids (eight species) and *Cladotaenia globifera* (Batsch, 1786) (Paruterinidae) include hosts representing two different vertebrate classes (birds and fishes, mammals and fishes, and birds and mammals). Among birds, the highest tapeworm diversity is found in anseriforms (34 spp.), charadriiforms (18 spp.) and passeriforms (14 spp.) (Table 1).

**Table 1.** The number of tapeworm species in various bird orders in the Iberian Peninsula (Spain and Portugal), Slovakia, Poland and Finland. For source references, see Materials and methods. If a tapeworm species occurs in more than one bird order, it has been exluded from the data.

Order	Iberian Peninsula	Slovakia	Poland	Finland
Anseriformes	15	55	65	34
Galliformes	12	10	9	3
Gaviiformes	-	-	3	6
Podicipediformes	2	10	17	5
Pelecaniformes	-	-	2	1
Ciconiiformes	2	6	6	-
Accipitriformes	-	1	4	1
Gruiformes	3	6	2	1
Charadriiformes	32	18	32	18
Phoenicopteriformes	-	-	3	-
Columbiformes	10	1	-	1
Strigiformes	1	-	-	1
Caprimulgiformes	1	-	-	-
Apodiformes	6	-	1	2
Coraciiformes	1	-	-	-
Piciformes	-	1	2	3
Passeriformes	23	28	21	14

The checklist includes 30 tapeworm species and 96 parasite species/host species combinations (including the 30 “new” species) that have not been previously reported from Finland, marked as “Present study” in the references/source column. Four of the Finnish tapeworm species are sporadic imported parasites of humans and domestic animals not exhibiting natural transmission in Finland (see Discussion). Eight of the tapeworm species in the present checklist have been recorded only from the former territories of Finland, either from the Petsamo (Pechenga) region at the coast of the Arctic Ocean or from Karelia in the south-east of Finland.

The Finnish tapeworms represent seven orders and 18 families. As expected, the order Cyclophyllidea is the most diverse element of the Finnish cestode fauna (134 species or 80% of the total diversity), Hymenolepididae (61 spp.) being the most species-rich family.

**Figure 1.**
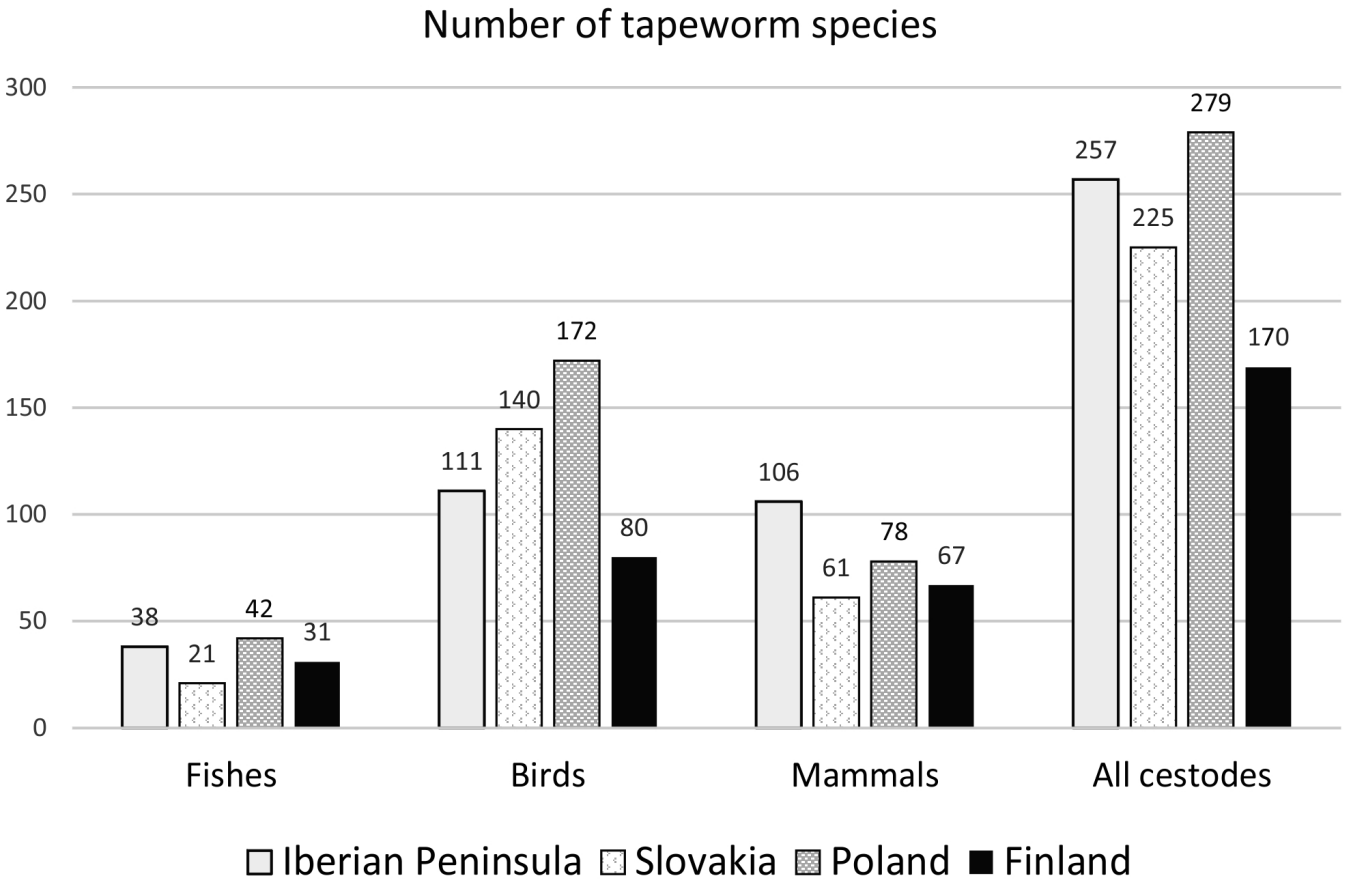
The number of tapeworm species of vertebrates (excluding amphibians and reptiles) in the Iberian Peninsula (Spain and Portugal), Slovakia, Poland anf Finland. For source references, see Materials and methods. The figures above columns show the exact number of species.

The total number of tapeworm species in Finland (170 spp.) is lower than the corresponding figure for the Iberian Peninsula (257 spp.), Slovakia (225 spp.) and Poland (279 spp.) (Fig. 1). The difference between Finland and the other three regions is particularly pronounced for birds, the Finnish species diversity being only 46–70% of the corresponding diversity in the other regions. Among birds, the tapeworm fauna of anseriforms, podicipediforms, charadriiforms and passeriforms is usually significantly lower in Finland than in the other parts of Europe (Table 1). The species diversity of tapeworms in galliform birds in Finland is also unexpectedly low, partly because no cestodes have been reported from Finnish chickens (*Gallus gallus domesticus*).

In addition, there is low tapeworm diversity in mammals in Finland (67 spp.) compared with that in the Iberian Peninsula (106 spp.). The latter difference is partly due to the presence of tapeworms of marine mammals in Spain and Portugal (12 spp.); such tapeworms are not known from Finland, because the only regularly occurring and breeding marine mammals in Finland are seals (*Halichoerus grypus* and *Pusa hispida*), which do not carry host-specific tapeworms. However, Finnish seals accidentally carry fish-transmitted tapeworms of water birds and predatory fishes.

The holotypes of five species of tapeworms originate from Finland: *Schistocephalus cotti*, *Paranoplocephala jarrelli* Haukisalmi, Henttonen & Hardman, 2006, *Paranoplocephala kalelai* (Tenora, Haukisalmi & Henttonen, 1985), *Catenotaenia henttoneni* Haukisalmi & Tenora, 1993 and *Taenia arctos* Haukisalmi, Lavikainen, Laaksonen & Meri, 2011 (see Checklist for collection numbers). The MZH collection also includes a slide of *Bothrimonus nylandicus* Schneider, 1902 from Finland that is marked by Guido Schneider as “typ-ex”, although he did not designate a type specimen in his publication (Schneider 1902a). The date and locality of the specimen match with those given in the original description. Thefore, this specimen is identified as the holotype of *Bothrimonus nylandicus*, and given the collection number MZH 127096. *Bothrimonus nylandicus* is presently considered a junior synonym of *Diplocotyle olrikii* Krabbe, 1874 (see Burt and Sandeman 1969). In addition, two specimens in ethanol, clearly representing previously unknown syntypes of *Caryophyllaeides fennica* (Schneider, 1902) from Finland (MZH 127097), were located in the MZH collection (see Schneider 1902b).

## Discussion

### General characteristics of the tapeworm fauna of mammals in Finland

This section describes various features of the tapeworm fauna of shrews, rodents (particularly voles and lemmings) and carnivores in Finland. The mammalian tapeworms are among the most extensively studied parasites in Finland, and practically all of them have been subject to molecular systematic analysis of some form. By contrast, evidently no published DNA sequence data exist for tapeworms of fishes and birds from Finland, with the exception of *Caryophyllaeides fennica* (see Brabec et al. 2012, Scholz et al. 2014), *Diphyllobothrium ditremum* and *Diphyllobothrium latum* (see Wicht et al. 2010).

One of the main patterns emerging from the present data is the seemingly unexplained absence in (northern) Fennoscandia of several mammalian tapeworms that have extensive distributions in the Holarctic region or in Eurasia.

### Shrews

There are six species of shrews (Soricidae) in Finland, five species of *Sorex* and the water shrew *Neomys fodiens*. According to the present checklist, *Sorex* shrews have 15 species of tapeworms, most of them hymenolepidids, parasitizing shrews in the adult stage [this figure excludes *Dilepis undula* (Schrank, 1788) and *Polycercus* sp., parasites of birds that do not reach full size and maturity in shrews]. The smaller and scarcer species of *Sorex* shrews (*Sorex minutus* with 6 species, *Sorex caecutiens* with 12 species) have more depauperate tapeworm assemblages than the larger ones, particularly when compared with the numerically dominant *Sorex araneus* (with 15 species) (see also Haukisalmi 1989). However, their faunas are overlapping in the sense that all the tapeworms of the smaller shrews also parasitize the larger ones. The only (partial) deviation to this pattern may be *Staphylocystoides stefanskii* (Żarnowski, 1954), which has been found most frequently from the pygmy shrew *Staphylocystoides minutus* in Finland (one record from *Staphylocystoides araneus*). On the other hand, *Staphylocystoides stefanskii* is known to parasitize six species of *Sorex* in Eurasia (Binkienė et al. 2011). The tapeworm fauna of the smallest and scarcest *Sorex* species, the least shrew *Sorex minutissimus*, is unknown in Finland.

The tapeworm fauna of *Sorex* shrews in Finland is very similar to that found elsewhere in Europe and western Eurasia. In Europe, there are only two species that have not been found from Finland, that is, *Skrjabinacanthus jacutensis* Spasskii & Morozov, 1959 and *Soricinia soricis* (Baer, 1928). *Skrjabinacanthus jacutensis* is a rare parasite of *Sorex* shrews with an extensive but very patchy distribution in Eurasia (Binkienė et al. 2011). It is possible that it occurs in Finland, but has not been found yet because of its rarity. The apparent absence of *Sorex soricis* in Finland may be due to the fact that it has been confused with *Soricinia infirma* (Żarnowski, 1955) (see Karpenko 1999).

Among the tapeworms of *Sorex* shrews, only *Spasskylepis ovaluteri* Schaldybin, 1964 can be regarded as a northern species; according to Binkienė et al. (2011) it has not been reported further south than Belarus in Europe, and it seems to have a northern distribution also elsewhere in Eurasia.

The molecular systematic analysis of Haukisalmi et al. (2010b) indicated that there is a *Ditestolepis* species in the taiga shrew *Sorex isodon* in Finland that is distinct from the type species *Ditestolepis diaphana* (Cholodkovsky, 1906) and related species representing other genera. Because there should not be other *Ditestolepis* species in Europe or western Eurasia (Binkienė et al. 2011), the cestode from *Sorex isodon* may be a previously unknown species. Alternatively, it may one of the poorly known *Ditestolepis* species described from Japan (see the Global Cestode Database; Caira et al. 2012).

The water shrews of the genus *Neomys* have an almost entirely separate tapeworm fauna when compared with the genus *Sorex*, although there is a number of scattered records of *Sorex* tapeworms parasitizing *Neomys* shrews (Binkienė et al. 2011). The tapeworm fauna of *Neomys fodiens* and *Neomys anomalus* in Europe comprise 15 species, all of them hymenolepidids (Binkienė et al. 2011, 2015), whereas only two tapeworm species are known from *Neomys fodiens* in Finland. One of these is typically a parasite of *Sorex* shrews [*Vigisolepis spinulosa* (Cholodkovsky, 1906)], and the other (*Polycercus* sp.) is a parasite of birds that accidentally infects shrews and other mammals (reported also from the raccoon dog *Nyctereutes procyonoides* in the present checklist). The specific identity of *Vigisolepis spinulosa* from the water shrew has been confirmed by DNA sequences (Haukisalmi et al. 2010b).

The apparent absence of host-specific tapeworms of *Neomys* in Finland could be due to biased sampling of water shrews and restricted distribution of freshwater amphipod crustaceans (Segerstråle 1954), the intermediate hosts of tapeworms of water shrews (Georgiev et al. 2006). The absence of host-specific tapeworms in *Neomys* in Finland seems to follow the general pattern for other parts of the northern Europe (Binkienė et al. 2011). Binkienė et al. (2011) suggested that the reason for the absence or extreme rarity of host-specific tapeworms in *Neomys* in the north is the low abundance of the definitive hosts. However, the restricted/patchy distribution of the amphipod intermediate hosts and their low numbers in the diet of water shrews seems to be an equally plausible explanation.

### Rodents (voles and lemmings)

Finland has a relatively diverse fauna of arvicoline rodents (Cricetidae), consisting of nine species of voles, including the introduced muskrat *Ondatra zibethicus*, and two species of lemmings.

In Finland, voles and lemmings have ten species of tapeworms parasitic in the adult stage, eight of them anoplocephalids, one catenotaeniid and one hymenolepidid cestode. The Finnish/northern European tapeworm fauna of arvicoline rodents can be classified into three main types: “endemics” of northenmost Europe (two species), species with a Holarctic distribution (one species) and species with extensive European/western Eurasian distribution (seven species).

*Paranoplocephala kalelai* (Tenora, Haukisalmi & Henttonen, 1985) and *Lemminia fellmani* (Haukisalmi & Henttonen, 2001), parasitizing voles of the genus *Myodes* (particularly the grey-sided vole *Myodes rufocanus*) and the Norwegian lemming *Lemmus lemmus*, respectively, appear to have distributions restricted to northern Fennoscandia. Based on the present knowledge, these species could be classified as the only endemic tapeworms of northern Europe.

The restricted distribution of *Paranoplocephala kalelai* seems curious, because its primary definitive host (*Myodes rufocanus*) has a continent-wide distribution in northern Eurasia. It is possible that *Paranoplocephala kalelai* has been misidentifed in earlier studies. For example, the extensive faunistical study of mammalian helminths in the north-west of the Ural mountains (Yushkov 1995) lists *Aprostatandrya macrocephala* (Douthitt, 1915), *Aprostatandrya caucasica* (Kirshenblat, 1938) and *Paranoplocephala omphalodes* (Hermann, 1783) as parasites of the grey-sided vole [the valid name of *Aprostatandrya macrocephala* is *Paranoplocephala macrocephala* (Douthitt, 1915) and *Aprostatandrya caucasica* is considered a junior synonym of *Paranoplocephala omphalodes*; see Haukisalmi et al. 2014]. Of these species, *Paranoplocephala macrocephala* is morphologically rather similar to *Paranoplocephala kalelai* (see Tenora et al. 1985a, Haukisalmi et al. 2007) and may have been confused with the latter. It is now known that *Paranoplocephala macrocephala* has a strictly North American distribution, parasitizing voles of the genus *Microtus* and geomyid rodents there (Haukisalmi and Henttonen 2003, Haukisalmi et al. 2004), although this name still appears as a parasite of arvicoline rodents in Eurasia. Thus, the true distribution of *Paranoplocephala kalelai* remains to be verified, but, based on the collections of the Beringian Coevolution Project (Hoberg et al. 2003, Cook et al. 2005), it does not occur in *Myodes rufocanus* in easternmost Siberia (Chukotka Peninsula and adjacent regions).

If the restricted northern distribution of *Paranoplocephala kalelai* is found to be real, this would support the idea that *Paranoplocephala kalelai* has diverged as a result of a host shift from a northern European *Microtus* lineage (most likely *Myodes oeconomus*) to the Fennoscandian subclade of *Myodes rufocanus* after its divergence from the Siberian *Myodes rufocanus* populations (Cook et al. 2004, Haukisalmi et al. 2007). This scenario is supported by two phylogenetic/phylogeographic analyses on tapeworms of the genus *Paranoplocephala* (see Haukisalmi et al. 2004, 2007).

*Lemminia fellmani* is known only from the Norwegian lemming *Lemminia lemmus* (a Fennoscandian endemic) from the mountains of southern Norway (Finse, type locality) and from northern Finland (Lapland) (Haukisalmi and Henttonen 2001). However, a morphologically and genetically related, congeneric cestode occurs in *Lemmus trimucronatus* is Alaska (Haukisalmi et al. 2010b), but it is uncertain if it is conspecific with *Lemminia fellmani*. No tapeworms have been found from the wood lemming *Myopus schisticolor* in Finland, although *Lemminia gubanovi* (Gulyaev & Krivopalov, 2003) occurs in this host in eastern Siberia (Gulyaev and Krivopalov 2003).

*Paranoplocephala jarrelli* Haukisalmi, Henttonen & Hardman, 2006 is known to parasitize the tundra/root vole *Microtus oeconomus* (and accidentally other *Microtus* species) from northern Finland to Alaska (Haukisalmi et al. 2004), therefore being the only tapeworm of Finnish rodents to have a Holarctic distribution, with the possible exception of *Lemminia fellmani* (above). The conspecificity of *Paranoplocephala jarrelli* populations in northern Finland, Hungary, the Russian Far East (Magadan) and Alaska has been verified by molecular methods (Haukisalmi et al. 2004).

Among the seven Finnish rodent tapeworms with an extensive European/western Eurasian distribution, Anoplocephaloides cf. dentata (Galli-Valerio, 1905), Microcephaloides cf. variabilis (Douthitt, 1915), *Microticola blanchardi* (Moniez, 1891), *Paranoplocephala omphalodes* (Hermann, 1783) and Hymenolepis (s.l.) asymmetrica Janicki, 1904 are primarily parasites of *Microtus* voles, *Catenotaenia henttoneni* is a parasite of *Myodes* voles (*Myodes glareolus* and *Myodes rutilus*) and *Eurotaenia gracilis* (Tenora & Murai, 1980) is a host-generalist parasite of voles and lemmings.

**Figure 2.**
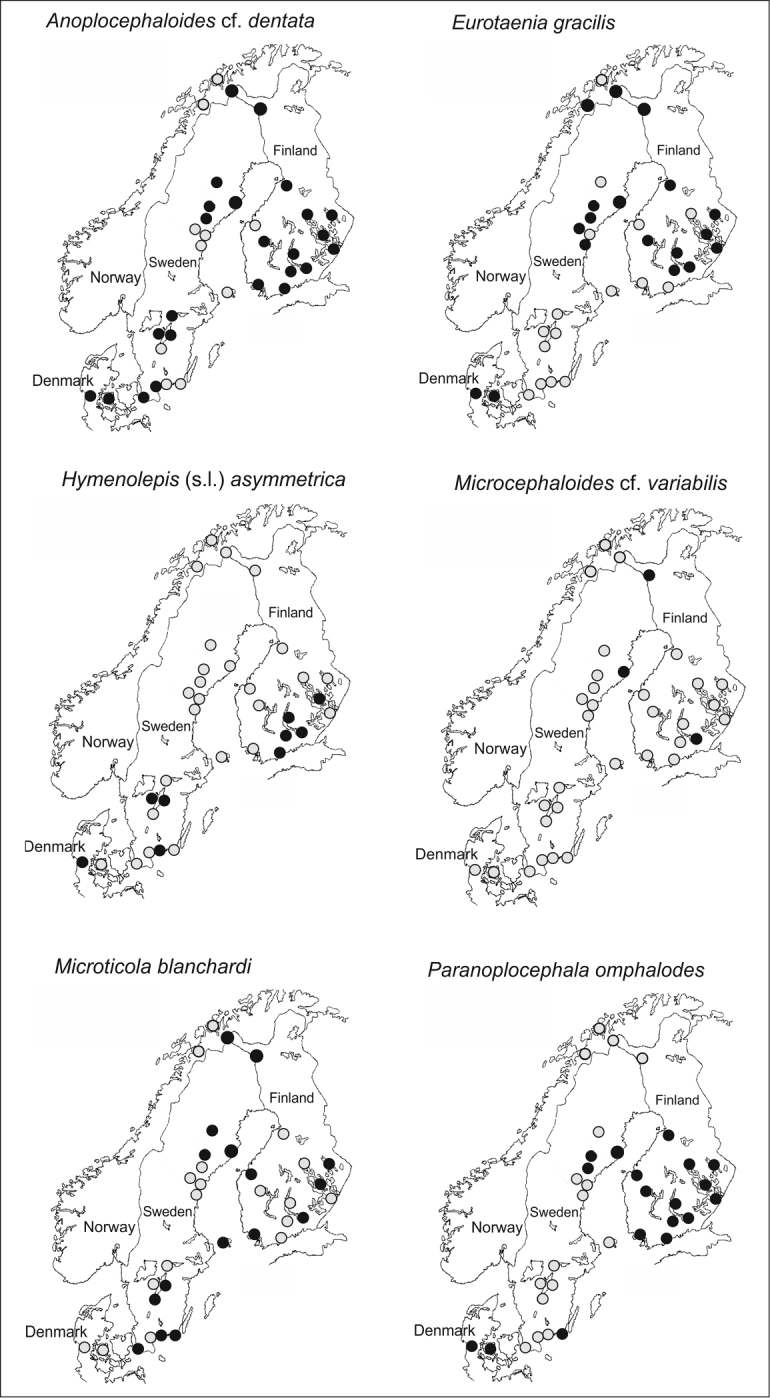
The geographical distribution of tapeworms of the field vole *Microtus agrestis* in Fennoscandia. All species except Hymenolepis (s.l.) asymmetrica (Hymenolepididae) represent the family Anoplocephalidae. Grey symbols, species absent; black symbols, species present. The number of voles examined for helminths in each locality varies considerably, but is usually more than ten (several hundred in Kilpisjärvi and Pallasjärvi in western Finnish Lapland).

Present data for the geographical distribution of tapeworms of the field vole *Microtus agrestis* in Fennoscandia (Fig. 2) show that the range of Anoplocephaloides cf. dentata, Microcephaloides cf. variabilis, *Microtus blanchardi* and *Eurotaenia gracilis* extends to the northenmost Fennoscandia, whereas *Paranoplocephala omphalodes* and *Hymenolepis asymmetrica* are absent from the truly northern regions. Of the latter two species, *Paranoplocephala omphalodes* has a more northerly distribution than *Hymenolepis asymmetrica*. The absence of these species from northernmost Finland is primarily based on nearly 40 years’ monitoring of arvicoline rodents and their helminths in western Finnish Lapland by H. Henttonen and coworkers, although extensive helminth datasets have been gathered also from other northern localities in Finland. The absence of these two species from the north seems peculiar, because their main definitive host (*Microtus agrestis*) occurs in the whole of the Fennoscandia, and is often the numerically dominant rodent species in open habitats throughout its range (Myllymäki et al. 1977).

It is noteworthy that no tapeworms of the genus *Arostrilepis* Mas-Coma & Tenora, 1997 (Hymenolepididae) have been reported from Finland or elsewhere from Fennoscandia, except for the finding of *Arostrilepis horrida* (von Linstow, 1901) from the bank vole *Myodes glareolus* from southern Norway (Baruš et al. 1977) and Russian Karelia (Mozgovoj et al. 1966). *Arostrilepis* species are ubiquitous parasites of arvicolines (and sporadically other rodents) in the Holarctic region, their range encompassing the central and southern Europe. Of the 12 valid species of *Arostrilepis*, at least eight occur in Eurasia (see the Global Cestode Database; Caira et al. 2012).

Another Holarctic tapeworm species evidently missing from Fennoscandia is *Anoplocephaloides lemmi* (Rausch, 1952), a parasite of lemmings of the genus *Lemmus* in northern Siberia and North America. The absence of this species seems real, because hundreds of Norwegian lemmings have been examined for helminths in Finnish Lapland and southern Norway by H. Henttonen and coworkers. It is hard to propose any general explanation for the absence of *Arostrilepis* species in most of Fennoscandia, but the absence of *Arostrilepis lemmi* and another host-specific, Holarctic tapeworm species of *Lemmus* spp. [*Arostrilepis beringiensis* (Kontrimavichus & Smirnova, 1991)] may be the result of the severe population bottle-neck experienced by *Lemmus lemmus* in Fennoscandia during the the last glacial maximum (Fedorov and Stenseth 2001, Haukisalmi and Henttonen 2001, Haukisalmi et al. in press).

*Hymenolepis diminuta* (Rudolphi, 1819) (a parasite of *Rattus* spp.) and *Hymenolepis hibernia* Montgomery, Montgomery & Dunn, 1987 (a parasite of *Apodemus* spp.) may also be listed as “missing” species, although there do not exist extensive helminthological studies for rats in Finland. The unverified record of *Hymenolepis* “*diminuta*” from *Apodemus flavicollis* (Raitis 1968; no voucher specimen exists), may, however, represent the latter tapeworm species.

### Carnivores

There are 14 species of terrestrial carnivores in Finland. The present study lists 17 tapeworm species parasitizing carnivores in the adult stage, Taeniidae (nine species) being the dominant element of the fauna. However, the taeniid fauna of Finnish carnivores should also include two additional species, *Taenia martis* and *Versteria mustelae* (parasites of mustelids), which have been found so far only as metacestodes from rodents. The metacestode of the latter species has also been found unexpectedly from the otter *Lutra lutra*. There are no published studies on tapeworms of mustelids in Finland.

Five of the Finnish carnivore tapeworms [*Dipylidium caninum* (Linnaeus, 1758), *Taenia solium* (Linnaeus, 1758), *Echinococcus equinus*, *Echinococcus granulosus* *s.s.*, *Echinoccus multilocularis* Leuckart, 1863] are clearly imported parasites that are not transmitted in Finland. The identification of recent imported infections of taeniid metacestodes in humans is based on DNA sequences (Lavikainen 2005, A. Lavikainen, unpubl.).

*Echinoccus multilocularis* is one of the tapeworm species that is mysteriously absent from Finland, although it has a Holarctic distribution and the definitive hosts (red fox *Vulpes vulpes* and other canids, including the raccoon dog) and intermediate hosts (rodents) are present in Finland. In addition, *Taenia crassiceps* (Zeder, 1800), a parasite of foxes that occurs basically throughout the Holarctic region, has not been found in Finland despite very extensive long-term studies on helminths of rodents (intermediate hosts of *Taenia crassiceps*) in Finland (H. Henttonen et al., unpublished). The absence of *Echinoccus multilocularis* and *Taenia crassiceps* may due to the fact that the density of the red fox, their primary definitive host, is below an (unknown) critical density for successful transmission of the parasite, and/or due to the pronounced density fluctuations of arvicoline rodents in Finland (Henttonen and Haukisalmi 2000). However, *Echinoccus multilocularis* has recently appeared in Denmark and Sweden (Kapell and Saeed 2000, Osterman Lind et al. 2011, Wahlström et al. 2012), and is predicted to spread to Finland as well.

*Taenia pisiformis*, with canids (including dog) as definitive hosts and hares as intermediate hosts, has evidently disappeared from Finland. In the 1940–50s, *Taenia pisiformis* was still a very common parasite in the country, known as the “bladder worm disease” of hares (Lampio 1946, 1950). However, no metacestodes of *Taenia pisiformis* were found from hares in early 1980s (Soveri and Valtonen 1983), and a recent survey of *Taenia* tapeworms in wolves from Finland and Sweden based on molecular identification (Lavikainen et al. 2011) also failed to find it. It is clear that the hunters’ awareness of the transmission of the parasite (hare offal should not be fed to dogs) and anthelmintic teatment of hunting dogs have played a major role in the disappearence of this parasite, but do not completely explain it, because suitable wild hosts are still numerous in Finland.

Recently, molecular methods have had a revolutionary impact on taeniid systematics. For example, the application of DNA based methods has enabled distinction of more or less cryptic, new species of *Taenia*, including *Taenia arctos*, a parasite of bears (definitive host) and cervids (intermediate hosts) in Finland, Alaska and Canada (Haukisalmi et al. 2011, Catalano et al. 2014). *Taenia arctos* had previously been confused with other *Taenia* species, mainly with *Taenia krabbei* Moniez, 1879, but it was found to be a genetically and biologically distinct entity (Lavikainen et al. 2010). Recently, another new species of *Taenia*, with the lynx (*Lynx lynx*) as a definitive host and cervids as intermediate hosts, has been found in Finland based on the molecular identification of adults and metacestodes (V. Haukisalmi, A. Lavikainen et al., unpubl.).

### Tapeworm diversity in different parts of Europe

One of the main patterns emerging from the present checklist and associated comparisons is that the tapeworm fauna of vertebrates in Finland is significantly less speciose than the corresponding fauna in other parts of Europe. The difference is mainly due to the low number of bird tapeworms in Finland.

Such a pronounced difference may be a real one or due to a number of confounding factors, including differences in latitude, available habitats (freshwater, marine, montane etc.), the number of host species present and the proportion of host species examined (adequately) for tapeworms. It is not possible to determine how these factors (interactively) determine the variation in tapeworm diversity in Europe, but the last factor probably explains most of the variation.

First, most of the tapeworms of vertebrates considered here have a wide European or western Eurasian (or more extensive) distribution, and are expected to occur in Fennoscandia, provided that their definitive and intermediate hosts are present. Therefore, latitude alone should not explain the differences in tapeworm diversity among regions. The availability of habitats is not a sufficient explanation either, because Finland is a long country stretching from the Baltic Sea (Gulf of Finland) to near the Arctic Ocean, and freshwater habitats (including thousands of lakes) are ubiquitous. Semi-montane landscape prevails in northern Finland (Lapland). The number of vertebrate host species certainly affects tapeworm diversity, and the high overall tapeworm diversity in the Iberian Peninsula is probably partly explained by this factor. However, there are no marked differences in vertebrate diversity between Slovakia, Poland and Finland, except that there are slightly fewer species of fishes and water birds in Slovakia because of the absence of marine habitats.

These patterns favour the idea that low tapeworm diversity in Finland is mainly due to insufficient sampling of vertebrates, particularly anseriform, podicipediform, charadriiform and passeriform birds. The tapeworm fauna of Poland, which is among the best known in Europe (Pojmańska et al. 2007), forms the most suitable model when predicting the true number of tapeworm species in Finland. The diversity of vertebrates is roughly equal in Poland and Finland, and there are no major faunistical differences either. In addition, Poland and Finland are both situated on the Baltic sea.

The tapeworms of fishes and mammals in Finland are relatively well known and the number of tapeworm species in these hosts is taken as such. In Poland, there are 172 species of tapeworms in birds, which is taken as the predicted number for the Finnish fauna. Based on this method, there should be ca. 270 species of tapeworms in Finland, instead of the 170 species listed in the present study.

## Appendix

Checklist of tapeworm species of vertebrates in Finland. Synonyms and misidentifications used in publications concerning the Finnish cestode fauna or in museum specimens have been indicated in brackets after the valid name. Abbreviations: MZH , Finnish Museum of Natural History, Helsinki . ZMUT , Zoological Museum of the University of Turku . * , record from the former Finnish territory (region specified in parentheses) . (l) , larval stage of tapeworm (metacestode) . HH , collected and identified by Heikki Henttonen and Voitto Haukisalmi . EVIRA , collected by specialists of the Finnish Food Safety Authority Evira . BMNH , British Museum of Natural History, London . USNPC , United States National Parasite Collection (presently housed in the National Museum of Natural History, Smithsonian Institution, Washington, D.C.) . MSB , Museum of Southwestern Biology, University of New Mexico, Albuquerque . HNHM , Hungarian Natural History Museum, Budapest .

**A.** Tapeworm species and their hosts.

Tapeworm taxa	Host species	References/source of specimens	Depositories/ collection numbers
**CARYOPHYLLIDEA**			
**Caryophyllaeidae**			
*Caryophyllaeus* Müller, 1787			
*Caryophyllaeus laticeps* (Pallas, 1781) [*Caryophyllaeus mutabilis* Rudolphi, 1802]	*Abramis brama*	Schneider 1902c, Pulkkinen and Valtonen 2012	MZH
	*Blicca bjoerkna*	Levander 1902, Schneider 1902c	MZH, ZMUT
	*Leuciscus leuciscus*	Present study (MZH)	MZH
	*Rutilus rutilus*	Valtonen et al. 1997	ZMUT
**Lytocestidae**			
*Caryophyllaeides* Nybelin, 1922			
*Caryophyllaeides fennica* (Schneider, 1902) [*Caryophyllaeus fennicus* Schneider, 1902]	*Alburnus alburnus*	Andersen and Valtonen 1990	-
	**Blicca bjoerkna* (Karelia)	Present study (MZH)	MZH
	*Carassius carassius*	Pulkkinen and Valtonen 2012	-
	*Leuciscus idus*	Schneider 1902c	MZH
	*Leuciscus leuciscus*	Andersen and Valtonen 1990	-
	*Rutilus rutilus*	Andersen and Valtonen 1990	ZMUT
	*Scardinius erythrophtalmus*	Schneider 1902b, Schneider 1902c	MZH 127097 (syntypes)
*Khawia* Hsü, 1935			
*Khawia rossittensis* (Szidat, 1937)	*Carassius carassius*	Gibson and Valtonen 1983	-
			
**SPATHEBOTHRIIDEA**			
**Acrobothriidae**			
*Cyathocephalus* Kessler, 1868			
*Cyathocephalus truncatus* (Pallas, 1781)	*Coregonus lavaretus*	Jääskeläinen 1910, Pulkkinen and Valtonen 2012	-
	*Salmo trutta*	Pulkkinen and Valtonen 2012	-
	**Thymallus thymallus* (Karelia)	Jääskeläinen 1910	MZH
*Diplocotyle* Krabbe, 1874			
*Diplocotyle olrikii* Krabbe, 1874 [*Bothrimonus nylandicus* Schneider, 1902, *Diplocotyle nylandica* (Schneider, 1902)]	*Gadus morhua*	Schneider 1902a, Pulkkinen and Valtonen 2012	-
	*Platichtys flesus*	Schneider 1902a	MZH 127096 (holotype of *Bothrimonus nylandicus*)
**DIPHYLLOBOTHRIIDEA**			
**Diphyllobothriidae**			
*Diphyllobothrium* Cobbold, 1878			
*Diphyllobothrium dendriticum* (Nitzsch, 1824) [*Diphyllobothrium norvegicum* Vik, 1957]	*Coregonus albula* (l)	Wikgren 1964, Valtonen et al. 1988	-
	*Coregonus lavaretus* (l)	Wikgren 1964, Pulkkinen and Valtonen 2012	-
	*Esox lucius* (l)	Pulkkinen and Valtonen 2012	-
	*Gasterosteus aculeatus* (l)	Valtonen and Julkunen 1995	-
	*Lota lota* (l)	Valtonen and Julkunen 1995	-
	*Triglopsis quadricornis* (l)	Valtonen and Julkunen 1995	-
	*Osmerus eperlanus* (l)	Pulkkinen and Valtonen 2012	-
	*Salmo salar* (l)	Valtonen et al. 2001	-
	*Salmo trutta* (l)	Pulkkinen and Valtonen 2012	-
	*Salvelinus alpinus* (l)	Pulkkinen and Valtonen 2012	-
*Diphyllobothrium ditremum* (Creplin, 1825) [*Diphyllobothrium osmeri* (von Linstow, 1878), *Diphyllobothrium vogeli* Kuhlow, 1953, *Bothriocephalus ditremus* Creplin, 1825]	*Gavia arctica*	Raitis 1968	ZMUT
	*Larus argentatus*	Raitis 1968	ZMUT
	*Mergus merganser*	Present study (HH)	-
	*Pusa hispida saimensis*	Sinisalo et al. 2003	-
	*Coregonus albula* (l)	Wikgren 1964, Valtonen et al. 1988	-
	*Coregonus lavaretus* (l)	Pulkkinen and Valtonen 2012	-
	*Gasterosteus aculeatus* (l)	Valtonen and Julkunen 1995	-
	*Lota lota* (l)	Valtonen and Julkunen 1995	-
	*Osmerus eperlanus* (l)	Wikgren 1964, Valtonen and Julkunen 1995	-
	*Pungitius pungitius* (l)	Pulkkinen and Valtonen 2012	-
	*Salmo trutta* (l)	Pulkkinen and Valtonen 2012	-
	*Salvelinus alpinus* (l)	Pulkkinen and Valtonen 2012	-
*Diphyllobothrium latum* (Linnaeus, 1758) [*Bothriocephalus latus* (Linnaeus, 1758), *Dibothriocephalus latus* (Linnaeus, 1758)]	*Canis lupus familiaris*	Oksanen 1972, Pullola et al. 2006	MZH
	*Homo sapiens*	Spöring 1747, Sievers 1905	MZH 44684
	*Vulpes vulpes*	Freeman 1964b	-
	*Esox lucius* (l)	Levander 1902, Pulkkinen and Valtonen 2012	MZH
	*Gymnocephalus cernuus* (l)	Levander 1902, Valtonen and Julkunen 1995	-
	*Lota lota* (l)	Valtonen and Julkunen 1995	-
	*Perca fluviatilis* (l)	Levander 1902, Valtonen et al. 1997	MZH
*Ligula* Bloch, 1782			
*Ligula intestinalis* (Linnaeus, 1758) [*Ligula simplicissima* Rudolphi, 1802]	*Gavia arctica*	Raitis 1968	MZH
	*Larus argentatus*	Present study (MZH)	MZH
	*Larus fuscus*	Present study (MZH)	MZH
	*Mergus merganser*	Present study (MZH)	MZH
	*Mergus serrator*	Schneider 1902c	MZH
	*Phalacrocorax carbo*	Levander 1927b, Lampio 1946	MZH
	*Podiceps cristatus*	Raitis 1968	MZH
	*Abramis brama* (l)	Pulkkinen and Valtonen 2012	MZH
	*Alburnus alburnus* (l)	Levander 1902, Pulkkinen and Valtonen 2012	MZH
	*Blicca bjoerkna*	Present study (MZH)	MZH
	*Leuciscus leuciscus* (l)	Pulkkinen and Valtonen 2012	MZH
	*Perca fluviatilis* (l)	Valtonen et al. 1997	MZH
	*Phoxinus phoxinus* (l)	Present study (MZH)	MZH
	*Rutilus rutilus* (l)	Valtonen et al. 1997	MZH
*Schistocephalus* Creplin, 1829			
*Schistocephalus cotti* Chubb, Seppälä, Lüscher, Milinski & Valtonen, 2006	*Cottus gobio* (l)	Chubb et al. 2006, Pulkkinen and Valtonen 2012	BMNH 2006.1.5.1 (holotype), BMNH 2006.1.5.2–7 (paratypes)
*Schistocephalus pungitii* Dubinina, 1959 [*Schistocephalus dimorphus* Creplin, 1829, *Schistocephalus gasterostei* (Fabricius, 1780), *Schistocephalus solidus* (Müller, 1776)]	*Pungitius pungitius* (l)	Schneider 1902c, Valtonen et al. 2001	MZH
*Schistocephalus solidus* (Müller, 1776) [*Schistocephalus gasterostei* (Fabricius, 1780)]	*Arenaria interpres*	Levander 1927a	-
	*Bucephala clangula*	Raitis 1968	ZMUT
	*Mergus serrator*	Schneider 1902c, Raitis 1968	ZMUT
	*Sterna hirundo*	Lemmetyinen and Raitis 1972	-
	*Sterna paradisaea*	Lemmetyinen and Raitis 1972	-
	*Pusa hispida botnica*	Chubb et al. 1995	-
	*Gasterosteus aculeatus* (l)	Schneider 1902c, Valtonen and Julkunen 1995	MZH
*Spirometra* Faust, Campbell & Kellogg, 1929			
*Spirometra* sp. [*Bothriocephalus felis* Creplin, 1852, *Bothriocephalus decipiens* Railliet, 1866]	*Lynx lynx*	Schneider 1906, Lavikainen et al. 2013, R. Kuchta & A. Lavikainen, unpubl.	-
**BOTHRIOCEPHALIDEA**			
**Bothriocephalidae**			
*Bothriocephalus* Rudolphi, 1808			
*Bothriocephalus claviceps* (Goeze, 1782)	*Anguilla anguilla*	Schneider 1902c	MZH
*Bothriocephalus scorpii* (Müller, 1776) [*Bothriocephalus punctatus* (Rudolphi, 1802)]	*Myoxocephalus scorpius*	Schneider 1902c	MZH
	*Scophthalmus maximus*	Schneider 1902c	MZH
	*Taurulus bubalis*	Schneider 1904	MZH
	*Triglopsis quadricornis*	Schneider 1904	MZH
**Triaenophoridae**			
*Abothrium* van Beneden, 1871			
*Abothrium gadi* van Beneden, 1871	**Gadus morhua* (Petsamo)	Raitis 1968	ZMUT
*Eubothrium* Nybelin, 1922			
*Eubothrium crassum* (Bloch, 1779) [*Abothrium crassum* (Bloch, 1779), *Bothriotaenia proboscidea* (Batsch, 1786), *Bothriocephalus proboscideus* (Batsch, 1786), *Dibothrium proboscideum* (Batsch, 1786)]	*Clupea harengus membras*	Schneider 1902c	MZH
	*Coregonus lavaretus*	Valtonen et al. 1988	-
	*Salmo salar*	Schneider 1902c, Andersen and Valtonen 1990	MZH
	*Salmo trutta*	Andersen and Valtonen 1990	MZH
*Eubothrium rugosum* (Batsch, 1786) [*Abothrium rugosum* (Batsch, 1786), *Bothriotaenia rugosa* (Batsch, 1786), *Bothriocephalus rugosus* (Batsch, 1786), *Dibothrium rugosum* (Batsch, 1786)]	*Lota lota*	Schneider 1904, Andersen and Valtonen 1990	MZH
*Eubothrium salvelini* (Schrank, 1790)	*Salmo trutta*	Pulkkinen and Valtonen 2012	MZH
	*Salvelinus alpinus*	Pulkkinen and Valtonen 2012	MZH
*Triaenophorus* Rudolphi, 1793			
*Triaenophorus crassus* Forel, 1868 [*Triaenophorus robustus* Olsson, 1893]	*Esox lucius*	Valtonen et al. 1989	MZH
	*Coregonus albula* (l)	Luther 1908, Valtonen et al. 1988	MZH
	*Coregonus lavaretus* (l)	Valtonen et al. 1988	MZH
	*Lampetra fluviatilis* (l)	Valtonen et al. 1989	-
	*Oncorhynchus mykiss* (l)	Pulkkinen and Valtonen 2012	-
	*Salvelinus alpinus* (l)	Pulkkinen and Valtonen 2012	-
	*Thymallus thymallus* (l)	Pulkkinen and Valtonen 2012	-
*Thymallus nodulosus* Sramek, 1901	*Esox lucius*	Schneider 1901, Valtonen et al. 1989	MZH
	*Esox lucius* (l)	Levander 1927c	MZH
	*Cottus gobio* (l)	Schneider 1904	-
	*Gasterosteus aculeatus* (l)	Valtonen et al. 1989	-
	*Gymnocephalus cernuus* (l)	Valtonen et al. 1989	-
	*Lota lota* (l)	Valtonen et al. 1989	MZH
	*Osmerus eperlanus* (l)	Valtonen et al. 1989	MZH
	*Perca fluviatilis* (l)	Schneider 1902c, Valtonen et al. 1989	MZH
	*Pungitius pungitius* (l)	Schneider 1902c, Valtonen et al. 1989	MZH
	*Salmo salar* (l)	Pulkkinen and Valtonen 2012	-
	*Salmo trutta* (l)	Pulkkinen and Valtonen 2012	-
	*Zoarces viviparus* (l)	Schneider 1904, Pulkkinen and Valtonen 2012	-
	*Pusa hispida saimensis*	Present study (MZH)	MZH
**PROTEOCEPHALIDEA**			
**Proteocephalidae**			
*Proteocephalus* Weinland, 1858			
*Proteocephalus ambiguus* (Dujardin, 1845) [*Ichthyotaenia ambigua* (Dujardin, 1845)]	*Pungitius pungitius*	Schneider 1905, Andersen and Valtonen 1990	-
*Proteocephalus cernuae* (Gmelin, 1790)	*Gymnocephalus cernuus*	Valtonen and Rintamäki 1989	ZMUT
*Proteocephalus filicollis* (Rudolphi, 1802)	*Gasterosteus aculeatus*	Schneider 1902c, Andersen and Valtonen 1990	-
*Proteocephalus gobiorum* Dogel & Bykhovskii, 1939	*Myoxocephalus scorpius*	Pulkkinen and Valtonen 2012	-
	*Pomatoschistus minutus*	Valtonen et al. 2001	MZH
	*Triglopsis quadricornis*	Pulkkinen and Valtonen 2012	ZMUT
*Proteocephalus longicollis* (Zeder, 1800) [*Taenia longicollis* Zeder, 1800, *Ichtyotaenia longicollis* (Zeder, 1800), *Proteocephalus exiguus* La Rue, 1911, *Pomatoschistus albulae* Freze & Kazakov, 1969]	*Coregonus albula*	Valtonen et al. 1988	-
	*Coregonus lavaretus*	Valtonen et al. 1988	MZH
	*Salvelinus alpinus*	Pulkkinen and Valtonen 2012	-
*Pomatoschistus macrocephalus* (Creplin, 1825) [*Ichtyotaenia macrocephala* (Creplin, 1825)]	*Anguilla anguilla*	Schneider 1902c	MZH
*Pomatoschistus percae* (Müller, 1780) [*Ichthyotaenia percae* (Müller, 1780), *Ichthyotaenia ocellata* (Rudolphi, 1802), *Ichthyotaenia filicollis* (Rudolphi, 1802)]	*Perca fluviatilis*	Schneider 1904, Valtonen and Rintamäki 1989	MZH
*Pomatoschistus tetrastomus* (Rudolphi, 1810) [*Pomatoschistus longicollis* (Zeder, 1800)]	*Osmerus eperlanus*	Andersen and Valtonen 1990	-
*Pomatoschistus thymalli* (Annenkova-Khlopina, 1923)	*Thymallus thymallus*	Present study (HH)	MZH
*Pomatoschistus torulosus* (Batsch, 1786) [*Taenia torulosa* Batsch, 1786, *Ichthyotaenia torulosa* (Batsch, 1786)]	*Abramis ballerus*	Present study (MZH)	MZH
	*Alburnus alburnus*	Present study (MZH)	MZH
	*Leuciscus idus*	Schneider 1902c	MZH
	*Leuciscus leuciscus*	Valtonen et al. 2001	-
	*Rutilus rutilus*	Valtonen et al. 1997	-
*Glanitaenia* de Chambrier, Zehnder, Vaucher & Mariaux, 2004			
*Glanitaenia osculata* (Goeze, 1782) [*Ichtyotaenia osculata* (Goeze, 1782)]	**Silurus glanis* (Karelia)	Present study (MZH)	MZH
**TETRABOTHRIIDEA**			
**Tetrabothriidae**			
*Tetrabothrius* Rudolphi, 1819			
*Tetrabothrius macrocephalus* (Rudolphi, 1810) [*Bothriocephalus macrocephalus* Rudolphi, 1810]	*Cepphus grylle*	Raitis 1968	ZMUT
	*Gavia stellata*	Present study (MZH, ZMUT)	MZH, ZMUT
	*Podiceps cristatus*	Raitis 1968	ZMUT
	*Uria aalge*	Present study (MZH)	MZH
*Tetrabothrius mawsoni* Johnston, 1937 [*Tetrabothrius cylindraceus* (Rudolphi, 1819)]	*Larus argentatus*	Raitis 1968	ZMUT
	*Podiceps cristatus*	Raitis 1968	ZMUT
	*Gavia stellata*	Raitis 1968	ZMUT
**CYCLOPHYLLIDEA**			
**Anoplocephalidae**			
*Anoplocephala* Blanchard, 1848			
*Anoplocephala perfoliata* (Goeze, 1782) [*Taenia perfoliata* Goeze, 1782]	*Equus caballus*	Saari and Nikander 1992	MZH
*Anoplocephaloides* Baer, 1923			
Anoplocephaloides cf. dentata (Galli-Valerio, 1905)	*Arvicola amphibius*	Present study (HH)	MZH
	*Lemmus lemmus*	Present study (HH)	MZH
	*Microtus agrestis*	Tenora et al. 1986b, Haukisalmi et al. 2009a	USNPC 95648, 97613–97615, MZH
	*Microtus oeconomus*	Tenora et al. 1986b, Haukisalmi et al. 2009a	USNPC 97612, 97616, 107977–107979, 107999, MZH
	*Myodes rufocanus*	Tenora et al. 1986b, Haukisalmi et al. 1987	MZH
*Eurotaenia* Haukisalmi, Hardman, Hoberg & Henttonen, 2014			
*Eurotaenia gracilis* (Tenora & Murai, 1980) [*Paranoplocephala gracilis* Tenora & Murai, 1980]	*Arvicola amphibius*	Present study (HH)	MZH
	*Lemmus lemmus*	Present study (HH)	MZH
	*Microtus agrestis*	Tenora et al. 1986a, Wickström et al. 2005	MZH
	*Microtus oeconomus*	Present study (HH)	MZH
	*Myodes glareolus*	Present study (HH)	MZH
	*Myodes rufocanus*	Tenora et al. 1986a	MZH
	*Myodes rutilus*	Present study (HH)	USNPC 107980, MZH (S)
*Lemminia* Haukisalmi, Hardman, Hoberg & Henttonen, 2014			
*Lemminia fellmani* (Haukisalmi & Henttonen, 2001) [*Paranoplocephala fellmani* Haukisalmi & Henttonen, 2001]	*Lemmus lemmus*	Haukisalmi and Henttonen 2001, Wickström et al. 2005	MZH 8406 (paratype)
*Microcephaloides* Haukisalmi, Hardman, Hardman, Rausch & Henttonen, 2008			
Microcephaloides cf. variabilis (Douthitt, 1915) [Anoplocephaloides cf. variabilis Douthitt, 1915]	*Microtus agrestis*	Tenora et al. 1986b, Haukisalmi et al. 2008	MSB Endo 74, MZH
	*Microtus oeconomus*	Haukisalmi et al. 2008	MSB Endo 72, 75, MZH
	*Myodes rufocanus*	Present study (HH)	MZH
*Microticola* Haukisalmi, Hardman, Hoberg & Henttonen, 2014			
*Microticola blanchardi* (Moniez, 1891) [Anoplocephaloides cf. blanchardi Moniez, 1891]	*Microtus agrestis*	Tenora et al. 1986b, Wickström et al. 2005	MZH
	*Microtus oeconomus*	Tenora et al. 1986b	MZH
*Moniezia* Blanchard, 1891			
*Moniezia expansa* (Rudolphi, 1810)	*Alces alces*	Nygrén and Wallén 2001	MZH
	**Ovis aries* (Karelia)	Pulkkinen 1932	-
*Moniezia benedeni* (Moniez, 1879)	*Bos taurus*	Present study	MZH
Moniezia cf. benedeni (Moniez, 1879), as *Moniezia* sp.	*Rangifer tarandus*	Wickström et al. 2005	MZH
*Mosgovoyia* Spasskii, 1951			
*Mosgovoyia pectinata* (Goeze, 1782) [*Cittotaenia pectinata* (Goeze, 1782)]	*Lepus europaeus*	Soveri and Valtonen 1983	
	*Lepus timidus*	Reuter 1882, Lampio 1946, Haukisalmi et al. 2010a	MZH
*Neoctenotaenia* Tenora, 1976			
*Neoctenotaenia ctenoides* (Railliet, 1890)	*Oryctolagus cuniculus*	Haukisalmi et al. 2010a	MZH
*Paranoplocephala* Lühe, 1910			
*Paranoplocephala omphalodes* (Hermann, 1783) [*Taenia omphalodes* Hermann, 1783, *Andrya omphalodes* (Hermann, 1783), *Andrya microti* Hansen, 1947]	*Arvicola amphibius*	Tenora et al. 1986a	MZH
	*Microtus agrestis*	Haukisalmi et al. 1994, 2004	USNPC 92584, MZH
	*Microtus levis*	Present study (HH)	MZH
	*Myodes glareolus*	Present study (HH)	MZH
*Paranoplocephala jarrelli* Haukisalmi, Henttonen & Hardman, 2006 [*Andrya microti* Hansen, 1947]	*Microtus oeconomus*	Haukisalmi et al. 2006, 2009b	USNPC 95640 (holotype), 95641 (paratype), 108003, HNHM 67468, MZH
*Paranoplocephala kalelai* (Tenora, Haukisalmi & Henttonen, 1985) [*Andrya kalelai* Tenora, Haukisalmi & Henttonen, 1985]	*Myodes glareolus*	Tenora et al. 1985a, Haukisalmi et al. 2007	USNPC 108001, 108002, MZH
	*Myodes rufocanus*	Tenora et al. 1985a, Haukisalmi et al. 2007	MZH 61034 (holotype), 61033, 61035 (paratypes)
	*Myodes rutilus*	Tenora et al. 1985a	MZH
**Catenotaeniidae**			
*Catenotaenia* Janicki, 1904			
*Catenotaenia henttoneni* Haukisalmi & Tenora, 1993 [*Catenotaenia cricetorum* Kirshenblat, 1949]	*Myodes glareolus*	Haukisalmi and Tenora 1993, Haukisalmi et al. 2010c	MZH 63142 (holotype), 63141 (paratype), USNPC 94886, 102583, 102585, 102582
	*Myodes rutilus*	Wiger et al. 1976, Haukisalmi and Tenora 1993, Haukisalmi et al. 2010c	USNPC 102584, 102586–102588, 107981, 107997, 107998, MZH
*Catenotaenia dendritica* (Goeze, 1782)	*Sciurus vulgaris*	Haukisalmi et al. 2010c	USNPC 102581, MZH
*Catenotaenia pusilla* (Goeze, 1782)	*Mus musculus*	Present study (HH)	-
*Skrjabinotaenia* Ahumyan, 1946			
*Skrjabinotaenia lobata* (Baer, 1925)	*Apodemus flavicollis*	Present study (HH)	MZH
**Davaineidae**			
*Ophryocotyle* Friis, 1870			
*Ophryocotyle proteus* Friis, 1870	**Limosa lapponica* (Petsamo)	Raitis 1968	ZMUT
*Paroniella* Fuhrmann, 1920			
*Paroniella urogalli* (Modeer, 1790) [*Taenia urogalli* Modeer, 1790, *Davainea urogalli* (Modeer, 1790)]	*Lagopus lagopus*	Isomursu et al. 2004	MZH
	*Lyrurus tetrix*	Lampio 1946, Isomursu et al. 2004	MZH
	*Perdix perdix*	Present study (MZH)	MZH
	*Tetrao urogallus*	Isomursu et al. 2004	MZH
	*Tetrastes bonasia*	Isomursu et al. 2004	MZH
*Raillietina* Fuhrmann, 1920			
*Raillietina frontina* (Dujardin, 1845) [*Davainea frontina* (Dujardin, 1845)]	*Dryocopus martius*	Raitis 1968	MZH, ZMUT
*Skrjabinia* Fuhrmann, 1920			
*Skrjabinotaenia cesticillus* (Molin, 1858)	*Lagopus lagopus*	Isomursu et al. 2004	MZH
	*Lyrurus tetrix*	Isomursu et al. 2004	-
	*Tetrao urogallus*	Isomursu et al. 2004	-
	*Tetrastes bonasia*	Isomursu et al. 2004	-
**Dilepididae**			
*Alcataenia* Spasskaya, 1971			
*Alcataenia campylacantha* (Krabbe, 1869) [*Anomotaenia campylacantha* (Krabbe, 1869), *Choanotaenia campylacantha* (Krabbe, 1869)]	**Cepphus grylle* (Petsamo)	Raitis 1968	MZH, ZMUT
*Alcataenia larina* (Krabbe, 1869) [*Anomotaenia larina* (Krabbe, 1869)]	*Larus canus*	Raitis 1968	ZMUT
*Angularella* Strand, 1928			
*Angularella* sp.	*Riparia riparia*	Raitis 1968	ZMUT
*Anomotaenia* Cohn, 1900			
*Anomotaenia arionis* (von Siebold, 1850) [*Choanotaenia arionis* (von Siebold, 1850)]	**Actitis hypoleucos* (Petsamo)	Raitis 1968	MZH
*Anomotaenia globulus* (Wedl, 1855)	*Scolopax rusticola*	Raitis 1968	ZMUT
*Anomotaenia microrhyncha* (Krabbe, 1869)	**Charadrius hiaticula* (Petsamo)	Raitis 1968	ZMUT
	**Philomachus pugnax* (Petsamo)	Raitis 1968	ZMUT
*Dictymetra* Clark, 1952			
*Dictymetra laevigata* (Rudolphi, 1819)	**Phalaropus lobatus* (Petsamo)		ZMUT
	*Numenius arquata*	Present study (ZMUT)	ZMUT
*Dilepis* Weinland, 1858			
*Dilepis undula* (Schrank, 1788) [*Taenia undulata* Rudolphi, 1810]	*Columba palumbus*	Raitis 1968	MZH, ZMUT
	*Corvus corone*	Raitis 1968	MZH, ZMUT
	*Pica pica*	Present study (MZH)	MZH
	*Turdus iliacus*	Raitis 1968	ZMUT
	*Turdus philomelos*	Present study (MZH)	MZH
	*Turdus pilaris*	Raitis 1968	MZH, ZMUT
	*Turdus viscivorus*	Present study (MZH)	MZH
	*Sorex araneus*	Haukisalmi 1989	-
*Fuhrmannolepis* Spasskii & Spasskaya, 1965			
*Fuhrmannolepis* sp.	*Scolopax rusticola*	Present study (ZMUT)	ZMUT
*Hepatocestus* Bona, 1994			
*Hepatocestus hepaticus* (Baer, 1932) [*Choanotaenia hepatica* (Baer, 1932)]	*Sorex araneus*	Vaucher, 1971, Haukisalmi 1989	-
*Hirundinicola* Birova-Volosinovicova, 1969			
*Hirundinicola parvirostris* (Krabbe, 1869)	**Delichon urbica* (Petsamo)	Raitis 1968	ZMUT
	*Hirundo rustica*	Raitis 1968	ZMUT
*Kowalewskiella* Baczynska, 1914			
*Kowalewskiella cingulifera* (Krabbe, 1869)	**Actitis hypoleucos* (Petsamo)	Raitis 1968	ZMUT
*Liga* Weinland, 1857			
*Liga crateriformis* (Goeze, 1782) [*Choanotaenia crateriformis* (Goeze, 1782), *Monopylidium crateriformis* (Goeze, 1782)]	*Dendrocopos leucotos*	Raitis 1968	ZMUT
	*Dendrocopos major*	Raitis 1968	MZH, ZMUT
	*Picus canus*	Raitis 1968	MZH, ZMUT
*Monocercus* Villot, 1882			
*Monocercus arionis* (von Siebold, 1850) [*Choatonotaenia crassiscolex* (von Linstow, 1890), *Molluscotaenia crassiscolex* (von Linstow, 1890)]	*Sorex araneus*	Vaucher, 1971, Haukisalmi 1989, Haukisalmi and Henttonen 1994	MZH
	*Sorex caecutiens*	Haukisalmi and Henttonen 1994	-
	*Sorex isodon*	Bugmyrin et al. 2003	-
	*Sorex minutus*	Haukisalmi 1989	-
*Monosertum* Bona, 1994			
*Monosertum parinum* (Dujardin, 1845) [*Choanotaenia parina* (Dujardin, 1845)]	*Fringilla montifringilla*	Raitis 1968	ZMUT
*Neoliga* Singh, 1952			
*Neoliga depressa* (von Siebold, 1836)	*Apus apus*	Present study (MZH)	MZH
*Neovalipora* Baer, 1962			
*Neovalipora parvispine* (Linton, 1927)	*Gavia stellata*	Present study (MZH)	MZH
*Nototaenia* Jones & Williams, 1967			
*Nototaenia brevis* (von Linstow, 1884) [*Amoebotaenia brevis* (von Linstow, 1884)]	*Pluvialis apricaria*	Raitis 1968	ZMUT
*Polycercus* Villot, 1883			
*Polycercus* sp.	*Neomys fodiens*	Present study (HH)	-
	*Nyctereutes procyonoides*	Present study (EVIRA)	-
*Rallitaenia* Spasskii & Spasskaya, 1975			
*Rallitaenia pyriformis* (Wedl, 1855)	*Crex crex*	Present study (MZH)	MZH
*Sacciuterina* Matevosyan, 1963			
*Sacciuterina paradoxa* (Rudolphi, 1802)	**Calidris alpina* (Petsamo)	Raitis 1968	ZMUT
	*Scolopax rusticola*	Present study (ZMUT)	ZMUT
*Sobolevitaenia* Spasskaya & Makarenko, 1965			
*Sobolevitaenia borealis* (Krabbe, 1869)	**Motacilla alba* (Petsamo)	Raitis 1968	ZMUT
*Spiniglans* Yamaguti, 1959			
*Spiniglans constricta* (Molin, 1858) [*Taenia constricta* Molin, 1858, *Anomotaenia constricta* (Molin, 1858), *Monopylidium constricta* (Molin, 1858)]	*Corvus corone*	Raitis 1968	MZH, ZMUT
*Spiniglans sharpiloi* Kornyushin, Salamatin, Greben, Georgiev, 2009	*Pica pica*	Present study (MZH)	MZH
*Trichocephaloidis* Sinitzin, 1896			
*Trichocephaloidis* sp.	*Tringa glareola*	Raitis 1968	ZMUT
**Dipylidiidae**			
*Dipylidium* Leuckart, 1863			
*Dipylidium caninum* (Linnaeus, 1758) [*Taenia cucumerina* Bloch, 1782]	*Canis lupus familiaris*	Oksanen 1972, Saari 1999	
**Hymenolepididae**			
*Aploparaksis* Clerc, 1903			
*Aploparaksis crassirostris* (Krabbe, 1869)	*Calidris alpina*	Raitis 1968	ZMUT
	*Limicola falcinella*	Present study (MZH)	MZH
	*Tringa glareola*	Raitis 1968	ZMUT
*Aploparaksis filum* (Goeze, 1782) s.l.	*Numenius arquata*	Raitis 1968	MZH, ZMUT
	*Scolopax rusticola*	Raitis 1968	MZH, ZMUT
	*Tringa glareola*	Present study (MZH)	MZH
*Aploparaksis furcigera* (Nitzsch in Rudolphi, 1819) [*Taenia rhomboidea* Dujardin, 1845, *Aploparaksis rhomboidea* (Dujardin, 1845)]	*Anas crecca*	Brglez and Valtonen 1986	-
	*Anas penelope*	Brglez and Valtonen 1986	-
	*Anas querquedula*	Brglez and Valtonen 1986	-
	*Anas platyrhynchos*	Brglez and Valtonen 1986	MZH
	*Aythya fuligula*	Valtonen and Brglez 1986	-
	*Bucephala clangula*	Raitis 1968	ZMUT
*Biglandatrium* Spasskaya, 1961			
*Biglandatrium biglandatrium* (Spasskaya, 1961)	*Gavia arctica*	Present study (MZH)	MZH
*Confluaria* Ablasov in Spasskaya, 1966			
*Confluaria furcifera* (Krabbe, 1869)	*Podiceps grisegena*	Present study (MZH)	MZH
*Confluaria multistriata* (Rudolphi, 1810)? [*Taenia multistriata* Rudolphi, 1810]	*Mergus merganser*	Present study (MZH)	MZH
*Confluaria pseudofurcifera* Vasileva, Georgiev & Genov, 2000 [*Hymenolepis furcifera* (Krabbe, 1869)]	*Podiceps cristatus*	Present study (MZH)	MZH
*Dicranotaenia* Railliet, 1892			
*Dicranotaenia coronula* (Dujardin, 1845) [*Hymenolepis coronula* (Dujardin, 1845)]	*Anas crecca*	Brglez and Valtonen 1986	-
	*Anas penelope*	Brglez and Valtonen 1986	-
	*Anas platyrhynchos*	Brglez and Valtonen 1986	MZH
	*Aythya fuligula*	Valtonen and Brglez 1986	-
	*Bucephala clangula*	Raitis 1968	ZMUT
	*Melanitta fusca*	Raitis 1968	MZH, ZMUT
*Diorchis* Clerc, 1903			
*Diorchis elisae* (Skrjabin, 1914)	*Anas crecca*	Brglez and Valtonen 1986	-
	*Anas platyrhynchos*	Brglez and Valtonen 1986	-
	*Anas querquedula*	Brglez and Valtonen 1986	-
	*Aythya fuligula*	Valtonen and Brglez 1986	-
*Diorchis inflata* (Rudolphi, 1819)	*Anas acuta*	Brglez and Valtonen 1986	-
	*Anas platyrhynchos*	Brglez and Valtonen 1986	-
*Diorchis stefanskii* Czaplinski, 1956	*Anas acuta*	Brglez and Valtonen 1986	-
	*Anas crecca*	Brglez and Valtonen 1986	-
	*Anas penelope*	Brglez and Valtonen 1986	-
	*Anas platyrhynchos*	Brglez and Valtonen 1986	-
	*Anas querquedula*	Brglez and Valtonen 1986	-
*Diorchis asiatica* Spasskii, 1963	*Anas penelope*	Brglez and Valtonen 1986	-
*Diorchis ransomi* Schultz, 1940	*Anas clypeata*	Brglez and Valtonen 1986	-
	*Anas crecca*	Brglez and Valtonen 1986	-
	*Anas platyrhynchos*	Brglez and Valtonen 1986	-
*Diploposthe* Jacobi, 1896			
*Diploposthe laevis* (Bloch, 1782)	*Anas penelope*	Brglez and Valtonen 1986	-
	*Aythya ferina*	Present study (MZH)	MZH
*Ditestolepis* Sołtys, 1952			
*Ditestolepis diaphana* (Cholodkovsky, 1906) [*Hymenolepis diaphana* Cholodkovsky, 1906]	*Sorex araneus*	Vaucher 1971, Haukisalmi 1989, Haukisalmi et al. 2010b	MZH
	*Sorex caecutiens*	Vaucher 1971, Haukisalmi 1989	-
	*Sorex isodon*	Bugmyrin et al. 2003	-
	*Sorex minutus*	Haukisalmi 1989	-
*Ditestolepis* sp.	*Sorex isodon*	Haukisalmi et al. 2010b	-
*Drepanidolepis* López-Neyra, 1942			
*Drepanidolepis anatina* (Krabbe, 1869) [*Hymenolepis anatina* (Krabbe, 1869)]	*Anas acuta*	Brglez and Valtonen 1986	-
	*Anas crecca*	Brglez and Valtonen 1986	-
	*Anas penelope*	Brglez and Valtonen 1986	-
	*Anas platyrhynchos*	Raitis 1968, Brglez and Valtonen 1986	ZMUT
*Drepanidolepis spinulosa* (Dubinina, 1953)	*Anas acuta*	Brglez and Valtonen 1986	-
	*Anas crecca*	Brglez and Valtonen 1986	-
	*Anas penelope*	Brglez and Valtonen 1986	-
	*Anas platyrhynchos*	Brglez and Valtonen 1986	-
	*Aythya fuligula*	Valtonen and Brglez 1986	-
*Drepanidolepis* sp. 1	*Melanitta fusca*	Present study (MZH)	MZH
*Drepanidolepis* sp. 2	*Melanitta fusca*	Present study (MZH)	MZH
*Drepanidotaenia* Railliet, 1892			
*Drepanidotaenia lanceolata* (Bloch, 1782)	*Anas penelope*	Brglez and Valtonen 1986	-
	*Anas querquedula*	Brglez and Valtonen 1986	-
*Dubininolepis* Spasskii & Spasskaya, 1954			
*Dubininolepis rostellata* (Abildgaard, 1790) [*Hymenolepis rostellata* (Abildgaard, 1790), *Hymenolepis capitellata* Railliet, 1899]	*Gavia arctica*	Raitis 1968	MZH, ZMUT
	*Gavia stellata*	Present study (ZMUT)	ZMUT
*Fimbriaria* Frölich, 1802			
*Fimbriaria fasciolaris* (Pallas, 1781) [*Taenia malleus* Goeze, 1782, *Fimbriaria plana* (von Linstow, 1905)]	*Anas acuta*	Brglez and Valtonen 1986	-
	*Anas clypeata*	Brglez and Valtonen 1986	-
	*Anas crecca*	Brglez and Valtonen 1986	-
	*Anas platyrhynchos*	Brglez and Valtonen 1986	MZH
	*Anas querquedula*	Brglez and Valtonen 1986	-
	*Aythya fuligula*	Valtonen and Brglez 1986	MZH
	*Mergus merganser*	Present study (MZH)	MZH
	**Mergus serrator* (Petsamo)	Raitis 1968	ZMUT
	**Somateria mollissima* (Petsamo)	Raitis 1968	ZMUT
*Gulyaevilepis* Kornienko & Binkiene, 2014			
*Gulyaevilepis tripartita* (Żarnowski, 1955) [*Hymenolepis tripartita* (Żarnowski, 1955), *Ditestolepis tripartita* (Żarnowski, 1955)]	*Sorex araneus*	Vaucher 1971, Haukisalmi 1989, Haukisalmi et al. 2010b	MZH
	*Sorex caecutiens*	Haukisalmi 1989	-
*Hymenolepis* Weinland, 1858			
Hymenolepis cf. diminuta (Rudolphi, 1819)	*Apodemus flavicollis*	Raitis 1968	ZMUT
Hymenolepis (s.l.) asymmetrica Janicki, 1904 [*Rodentolepis asymmetrica* (Janicki, 1904)]	*Microtus agrestis*	Haukisalmi et al. 1994	MZH
*Hymenolepis* (s.l.) sp.	*Lagopus lagopus*	Isomursu et al. 2004	-
	*Lyrurus tetrix*	Isomursu et al. 2004	-
	*Tetrao urogallus*	Isomursu et al. 2004	-
	*Tetrastes bonasia*	Isomursu et al. 2004	-
*Lineolepis* Spasskii, 1959			
*Lineolepis scutigera* (Dujardin, 1845) [*Hymenolepis scutigera* (Dujardin, 1845)]	*Sorex araneus*	Vaucher 1971, Haukisalmi 1989, Haukisalmi et al. 2010b	MZH
	*Sorex caecutiens*	Haukisalmi 1989	-
*Microsomacanthus* Lopez-Neyra, 1942			
*Microsomacanthus abortiva* (von Linstow, 1904)	*Anas acuta*	Brglez and Valtonen 1986	-
*Microsomacanthus arcuata* (Kowalewski, 1904)	*Anas acuta*	Brglez and Valtonen 1986	-
	*Anas clypeata*	Brglez and Valtonen 1986	-
	*Anas crecca*	Brglez and Valtonen 1986	-
	*Aythya fuligula*	Valtonen and Brglez 1986	-
*Microsomacanthus collaris* (Batsch, 1786) [*Hymenolepis collaris* (Batsch, 1786), *Myxolepis collaris* (Batsch, 1786), *Taenia sinuosa* Zeder, 1803, *Hymenolepis sinuosa* Railliet, 1899]	*Anas acuta*	Brglez and Valtonen 1986	-
	*Anas clypeata*	Brglez and Valtonen 1986	-
	*Anas crecca*	Raitis 1968, Brglez and Valtonen 1986	MZH, ZMUT
	*Anas platyrhynchos*	Raitis 1968, Brglez and Valtonen 1986	ZMUT
	*Aythya ferina*	Raitis 1968	ZMUT
*Microsomacanthus compressa* (Linton, 1892)	*Anas clypeata*	Brglez and Valtonen 1986	-
	*Anas crecca*	Brglez and Valtonen 1986	-
	*Anas penelope*	Brglez and Valtonen 1986	-
	*Aythya fuligula*	Valtonen and Brglez 1986	-
	*Aythya marila*	Present study (ZMUT)	ZMUT
*Microsomacanthus diorchis* (Fuhrmann, 1913)	*Somateria mollissima*	Present study (MZH)	MZH
*Microsomacanthus microsoma* (Creplin, 1829) [*Hymenolepis microsoma* (Creplin, 1829)]	*Somateria mollissima*	Raitis 1968	ZMUT
*Microsomacanthus paracompressa* (Czaplinski, 1956)	*Anas acuta*	Brglez and Valtonen 1986	-
	*Anas crecca*	Brglez and Valtonen 1986	-
	*Anas platyrhynchos*	Brglez and Valtonen 1986	-
	*Aythya fuligula*	Valtonen and Brglez 1986	-
*Microsomacanthus paramicrosoma* (Gasowska, 1931)	*Somateria mollissima*	Present study (MZH)	MZH
*Neoskrjabinolepis* Spasskii, 1947			
*Neoskrjabinolepis merkushevae* Kornienko & Binkienė, 2008	*Sorex araneus*	Present study (S. Kornienko & L. Kontrimavichus, unpubl.)	-
	*Sorex caecutiens*	Present study (S. Kornienko & L. Kontrimavichus, unpubl.)	-
*Neoskrjabinolepis schaldybini* Spasskii, 1947 [*Hymenolepis schaldybini* (Spasskii, 1947)]	*Sorex araneus*	Vaucher 1971, Haukisalmi 1989, Haukisalmi et al. 2010b	MZH
	*Sorex caecutiens*	Vaucher 1971, Haukisalmi 1989	MZH
	*Sorex isodon*	Present study (HH)	-
	*Sorex minutus*	Haukisalmi 1989	-
*Neoskrjabinolepis singularis* (Cholodkovsky, 1912) [*Hymenolepis singularis* Cholodkovsky, 1912]	*Sorex araneus*	Vaucher 1971, Haukisalmi 1989	-
	*Sorex caecutiens*	Haukisalmi 1989	-
*Nomadolepis* Makarikov, Gulyaev & Krivopalov, 2010			
*Nomadolepis* sp.	*Micromys minutus*	Haukisalmi et al. 2010b, Makarikov et al. 2015	
*Passerilepis* Spasskii & Spasskaya, 1954			
*Passerilepis crenata* (Goeze, 1782) [*Hymenolepis serpentulus* (Schrank, 1788)]	*Corvus corone*	Raitis 1968	ZMUT
	*Turdus iliacus*	Present study (MZH)	MZH
	*Turdus pilaris*	Present study (MZH)	MZH
	*Turdus viscivorus*	Present study (MZH)	MZH
*Passerilepis parina* (Fuhrmann, 1907)	*Parus major*	Present study (EVIRA)	MZH
*Parus stylosa* (Rudolphi, 1809) [*Taenia stylosa* Rudolphi, 1809]	*Pica pica*	Present study (MZH)	MZH
*Pseudobotrialepis* Schaldybin, 1957			
*Pseudobotrialepis globosoides* (Sołtys, 1954) [*Hymenolepis globosoides* (Soltys, 1954), *Dicranotaenia globosoides* Soltys, 1954]	*Sorex araneus*	Vaucher 1971, Haukisalmi 1989, Haukisalmi and Henttonen 1994	-
	*Sorex caecutiens*	Haukisalmi and Henttonen 1994	-
	*Sorex minutus*	Haukisalmi 1989, Haukisalmi et al. 2010b	MZH
*Retinometra* Spasskii, 1955			
*Retinometra macracanthos* (von Linstow, 1877)	*Anas acuta*	Brglez and Valtonen 1986	-
	*Anas penelope*	Brglez and Valtonen 1986	-
	*Anas platyrhynchos*	Brglez and Valtonen 1986	-
	*Aythya marila*	Present study (ZMUT)	ZMUT
*Rodentolepis* Spasskii, 1954			
*Rodentolepis fraterna* (Stiles, 1906)	*Apodemus flavicollis*	Present study (HH)	-
*Sobolevicanthus* Spasskii & Spasskaya, 1954			
*Sobolevicanthus dafilae* Polk, 1942	*Anas acuta*	Brglez and Valtonen 1986	-
	*Anas crecca*	Brglez and Valtonen 1986	-
	*Aythya fuligula*	Valtonen and Brglez 1986	-
*Sobolevicanthus octacanthus* (Krabbe, 1869)	*Anas crecca*	Brglez and Valtonen 1986	-
	*Anas platyrhynchos*	Brglez and Valtonen 1986	-
	*Anas querquedula*	Brglez and Valtonen 1986	-
	*Aythya fuligula*	Valtonen and Brglez 1986	-
*Sobolevicanthus gracilis* (Zeder, 1803) [*Hymenolepis gracilis* (Zeder, 1803)]	*Anas clypeata*	Brglez and Valtonen 1986	-
	*Anas crecca*	Raitis 1968, Brglez and Valtonen 1986	ZMUT
	*Anas platyrhynchos*	Brglez and Valtonen 1986	-
	*Aythya fuligula*	Valtonen and Brglez 1986	-
	**Mergus serrator* (Petsamo)	Raitis 1968	ZMUT
*Sobolevicanthus krabbeella* (Hughes, 1940)	*Anas crecca*	Brglez and Valtonen 1986	-
	*Aythya fuligula*	Valtonen and Brglez 1986	-
*Soricinia* Spasskii & Spasskaya, 1954			
*Soricinia infirma* (Żarnowski, 1955) [*Hymenolepis infirma* (Żarnowski, 1955), *Insectivorolepis infirma* Żarnowski, 1955]	*Sorex araneus*	Vaucher 1971, Haukisalmi 1989, Haukisalmi et al. 2010b	MZH
	*Sorex caecutiens*	Haukisalmi 1989	MZH
*Spasskylepis* Schaldybin, 1964			
*Spasskylepis ovaluteri* Schaldybin, 1964	*Sorex araneus*	Present study (HH)	-
	*Sorex caecutiens*	Haukisalmi et al. 2010b	-
*Staphylocystis* Villot,1877			
*Staphylocystis furcata* (Stieda, 1862) [*Hymenolepis furcata* (Stieda, 1862)]	*Sorex araneus*	Vaucher 1971, Haukisalmi 1989, Haukisalmi and Henttonen 1994, Haukisalmi et al. 2010b	MZH
	*Sorex caecutiens*	Haukisalmi and Henttonen 1994	-
*Staphylocystoides* Yamaguti, 1959			
*Staphylocystoides stefanskii* (Żarnowski, 1954)	*Sorex araneus*	Present study (HH)	-
	*Sorex minutus*	Haukisalmi et al. 2010b	-
	*Sorex* sp.	Vaucher, 1971	-
*Tschertkovilepis* Spassky & Spasskaya, 1954			
*Tschertkovilepis tenuirostris* (Rudolphi, 1819) [*Taenia tenuirostris* Rudolphi, 1819]	*Mergus merganser*	Present study (MZH)	MZH
*Urocystis* Villot, 1880			
*Urocystis prolifer* Villot, 1880 [*Hymenolepis prolifer* (Villot, 1880)]	*Sorex araneus*	Haukisalmi 1989, Haukisalmi et al. 2010b	MZH
*Vampirolepis* Spasskii, 1954			
*Vampirolepis* sp.	*Eptesicus nilssoni*	Haukisalmi et al. 2010b	-
*Variolepis* Spasskii & Spasskaya, 1954			
*Variolepis farciminosa* (Goeze, 1782) [*Hymenolepis farciminosa* (Goeze, 1782)]	*Sturnus vulgaris*	Present study (MZH)	MZH
			
*Vigisolepis* Matevosyan, 1945			
*Vigisolepis spinulosa* (Cholodkovsky, 1906) [*Hymenolepis spinulosa* Cholodkovsky, 1906]	*Sorex araneus*	Vaucher, 1971, Haukisalmi 1989, Haukisalmi et al. 2010b	MZH
	*Sorex caecutiens*	Haukisalmi 1989	-
	*Sorex isodon*	Present study (HH)	-
	*Sorex minutus*	Haukisalmi 1989	-
	*Neomys fodiens*	Present study (HH)	-
*Wardium* Mayhew, 1925			
*Wardium creplini* (Krabbe, 1869) [*Hymenolepis creplini* (Krabbe, 1869)]	*Anser fabalis*	Raitis 1968	ZMUT
*Wardoides* Spasskii, 1963			
*Wardoides nyrocae* (Yamaguti, 1935)	*Cygnus cygnus*	Present study (EVIRA)	MZH
			
**Linstowiidae**			
*Atriotaenia* Sandground, 1926			
*Atriotaenia incisa* (Railliet, 1899)	*Meles meles*	Present study (MZH)	MZH
**Mesocestoididae**			
*Mesocestoides* Vaillant, 1863			
*Mesocestoides lineatus* (Goeze, 1782)	*Canis lupus*	Present study (MZH)	MZH
	*Martes martes*	Present study (A. Lavikainen, unpubl.)	-
	*Meles meles*	Present study (EVIRA)	MZH
*Meles litteratus* (Batsch, 1786)	*Vulpes vulpes*	Freeman 1964a	-
*Mesocestoides* sp.	*Apodemus flavicollis* (l)	Present study (HH)	-
	*Microtus agrestis* (l)		-
	*Myodes glareolus* (l)	Present study (HH)	-
	*Myodes rufocanus* (l)	Present study (HH)	-
	*Myodes rutilus* (l)	Present study (HH)	-
	*Sorex araneus* (l)	Present study (HH)	-
**Paruterinidae**			
*Anonchotaenia* Cohn, 1900			
*Anonchotaenia globata* (von Linstow, 1879)	*Anthus trivialis*	Present study (MZH)	MZH
*Biuterina* Fuhrmann, 1902			
*Biuterina* sp.	*Lanius collurio*	Present study (MZH)	MZH
*Cladotaenia* Cohn, 1901			
*Cladotaenia globifera* (Batsch, 1786) [*Taenia cylindracea* Bloch, 1782, *Cladotaenia cylindracea* (Bloch, 1782)]	*Buteo buteo*	Present study (MZH)	MZH
	*Buteo lagopus*	Raitis 1968	ZMUT
	*Myodes glareolus* (l)	Tenora et al. 1983	-
*Notopentorchis* Burt, 1938			
*Notopentorchis cyathiformis* (Frölich, 1791) [*Taenia cyathiformis* Frölich, 1791]	*Apus apus*	Present study (MZH)	MZH
*Orthoskrjabinia* Spasskii, 1947			
*Orthoskrjabinia* sp.	*Picoides tridactylus*	Present study (MZH)	MZH
*Paruterina* Fuhrmann, 1906			
*Paruterina candelabraria* (Goeze, 1782)	*Aegolius funereus*	Present study (MZH)	MZH
	*Strix uralensis*	Present study (EVIRA)	MZH
*Paruterina parallelepipeda* (Rudolphi, 1810)	*Lanius collurio*	Raitis 1968	ZMUT
**Taeniidae**			
*Taenia* Linnaeus, 1758			
*Taenia arctos* Haukisalmi, Lavikainen, Laaksonen & Meri, 2011	*Ursus arctos*	Lavikainen et al. 2011, Haukisalmi et al. 2011	USNPC 104371 (holotype), 104372 (paratype), 104373–104375, MZH
	*Alces alces* (l)	Lavikainen et al. 2010	-
*Taenia hydatigena* Pallas, 1766 [*Cysticercus tenuicollis* Rudolphi, 1810]	*Canis lupus*	Lavikainen et al. 2011	MZH
	*Alces alces* (l)	Lampio 1946	MZH
	*Ovis aries* (l)	Raitis 1968, Lavikainen et al. 2008	ZMUT
	*Rangifer tarandus* (l)	Lavikainen et al. 2008	-
	*Sus scrofa*, domestic (l)	Present study (MZH)	MZH
*Taenia krabbei* Moniez, 1879 [*Cysticercus tarandi* Villot, 1883]	*Canis lupus*	Lavikainen et al. 2011	MZH
	*Rangifer tarandus* (l)	Rahkio and Korkeala 1989	-
*Taenia laticollis* Rudolphi, 1819	*Lynx lynx*	Lampio 1946, Lavikainen et al. 2013, Deksne et al. 2013	MZH
*Taenia martis* (Zeder, 1803)	*Myodes glareolus* (l)	Present study (MZH)	MZH
	*Myodes rutilus* (l)	Wiger et al. 1976	-
*Taenia pisiformis* (Bloch, 1780) [*Taenia serrata* Goeze, 1782, *Cysticercus pisiformis* Zeder, 1803]	*Canis lupus familiaris*	Lahermaa 1944, Lampio 1950	-
	*Lepus europaeus* (l)	Lampio 1946	-
	*Lepus timidus* (l)	Lahermaa 1944, Lampio 1946	MZH
*Taenia polyacantha* Leuckart, 1856	*Vulpes vulpes*	Freeman 1964a, Lavikainen et al. 2008	-
	*Microtus levis* (l)	Present study (HH)	-
	*Microtus oeconomus* (l)	Lavikainen et al. 2008	-
	*Myodes glareolus* (l)	Haukisalmi and Henttonen 1993, Lavikainen et al. 2008	USNPC 94887, 108005
	*Myodes rutilus* (l)	Wiger et al. 1976	-
*Taenia saginata* Goeze, 1782 [*Cysticercus bovis* Cobbold, 1866, *Cysticercus inermis*, *Taenia mediocanellata* Küchenmeister, 1852]	*Homo sapiens*	Pippingsköld 1869, Sievers 1905	MZH
	*Bos taurus* (l)	Niemiaho 1964	MZH
*Taenia solium* Linnaeus, 1758 [*Cysticercus cellulosae* (Gmelin, 1790)]	*Homo sapiens*	Sievers 1903, 1905	MZH
	*Homo sapiens* (l)	Saltzman 1868, Sievers 1905	-
*Taenia* sp.	*Lynx lynx*	Lavikainen et al. 2013	MZH
	*Alces alces* (l)	Present study (EVIRA)	MZH
	*Capreolus capreolus* (l)	Present study (EVIRA)	MZH
*Hydatigera* Lamarck, 1816			
*Hydatigera taeniaeformis* (Batsch, 1786) s.l. [*Taenia taeniaeformis* Batsch, 1786, *Taenia crassicollis* Rudolphi, 1810, *Cysticercus fasciolaris* Rudolphi, 1808]	*Felis silvestris catus*	Lavikainen et al. 2008	MZH
	*Lynx lynx*	Lavikainen et al. 2013	MZH
	*Apodemus flavicollis* (l)	Tenora et al. 1983	-
	*Microtus agrestis* (l)	Tenora et al. 1983, Haukisalmi et al. 1994	-
	*Myodes rutilus* (l)	Wiger et al. 1976	-
	*Ondatra zibethicus* (l)	Helminen 1957, Tenora et al. 1985b	MZH
	*Rattus norvegicus* (l)	Present study (MZH)	MZH
*Versteria* Nakao, Lavikainen, Iwaki, Haukisalmi, Konyaev, Oku, Okamoto & Ito, 2013			
*Versteria mustelae* (Gmelin, 1790) [*Taenia mustelae* Gmelin, 1790, *Taenia tenuicollis* Rudolphi, 1819]	*Lutra lutra* (l)	Present study (EVIRA)	-
	*Microtus agrestis* (l)	Tenora et al. 1983	-
	*Microtus oeconomus* (l)	Tenora et al. 1983	-
	*Myodes glareolus* (l)	Tenora et al. 1983, Lavikainen et al. 2008	USNPC 108061, 108070, 108076, 108080, 108085, 108092, 108104, 108111
	*Myodes rufocanus* (l)	Tenora et al. 1983, Lavikainen et al. 2008	-
	*Myodes rutilus* (l)	Tenora et al. 1983, Lavikainen et al. 2008	-
*Echinococcus* Rudolphi, 1801			
*Echinococcus canadensis* (Cameron, 1960) [*Echinococcus granulosus* (Batsch, 1786)]	*Canis lupus*	Hirvelä-Koski et al. 2003	-
	*Alces alces* (l)	Lavikainen et al. 2003	-
	*Rangifer tarandus* (l)	Lavikainen et al. 2003	-
	*Homo sapiens* (l)	Oksanen and Lavikainen in press, Hämäläinen et al., unpubl.	
*Echinococcus equinus* Williams & Sweatman, 1963	*Equus caballus* (l)	Saarma et al. 2009	-
*Echinococcus granulosus* (Batsch, 1786) s.l.	*Homo sapiens* (l)	Sievers 1889, 1905, Fagerlund 1890, Schulten 1890, Faltin 1914	MZH
*Echinococcus granulosus* (Batsch, 1786) s.s.	*Homo sapiens* (l)	Lavikainen 2005	-
*Echinococcus multilocularis* Leuckart, 1863	*Homo sapiens* (l)	Present study (A. Lavikainen, unpubl.)	-

**B.** Host species and their tapeworms

**CYCLOSTOMATA (jawless fishes, ympyräsuiset)**
**Petromyzontidae (northern lampreys, nahkiaiset)**
*Lampetra fluviatilis* (lamprey, nahkiainen)
*Triaenophorus crassus* (l)
**ACTINOPTERYGII (ray-finned fishes, viuhkaeväiset kalat)**
**Siluridae (catfishes, monnit)**
*Silurus glanis* (wels catfish, monni)
**Glanitaenia osculata*
**Percidae (percids, ahvenet)**
*Gymnocephalus cernuus* (ruffe, kiiski)
*Diphyllobothrium latum* (l)
*Triaenophorus nodulosus* (l)
*Proteocephalus cernuae*
*Perca fluviatilis* (European perch, ahven)
*Diphyllobothrium latum* (l)
*Ligula intestinalis* (l)
*Triaenophorus nodulosus* (l)
*Proteocephalus percae*
**Zoarcidae (eelpouts, kivinilkat)**
*Zoarces viviparus* (viviparous eelpout, kivinilkka)
*Triaenophorus nodulosus* (l)
**Gobiidae (gobies, tokot)**
*Pomatoschistus minutus* (sand goby, hietatokko)
*Proteocephalus gobiorum*
**Anguillidae (freshwater eels, ankeriaat)**
*Anguilla anguilla* (European eel, ankerias)
*Bothriocephalus claviceps*
*Proteocephalus macrocephalus*
**Esocidae (pikes, hauet)**
*Esox lucius* (northern pike, hauki)
*Diphyllobothrium dendriticum* (l)
*Diphyllobothrium latum* (l)
*Triaenophorus crassus*
*Triaenophorus nodulosus*/*Triaenophorus nodulosus* (l)
**Pleuronectidae (flounders, oikeasilmäkampelat)**
*Platichtys flesus* (European flounder, kampela)
*Diplocotyle olrikii*
**Scophthalmidae (turbots, piikkikampelat)**
*Scophthalmus maximus* (turbot, piikkikampela)
*Bothriocephalus scorpii*
**Cyprinidae (cyprinids, särkikalat)**
*Abramis brama* (bream, lahna)
*Caryophyllaeus laticeps*
*Ligula intestinalis* (l)
*Abramis ballerus* (blue bream, sulkava)
*Proteocephalus torulosus*
*Alburnus alburnus* (common bleak, salakka)
*Caryophyllaeides fennica*
*Ligula intestinalis* (l)
*Proteocephalus torulosus*
*Blicca bjoerkna* (silver bream, pasuri)
**Caryophyllaeides fennica*
*Caryophyllaeus laticeps*
*Ligula intestinalis* (l)
*Carassius carassius* (crucian carp, ruutana)
*Caryophyllaeides fennica*
*Khawia rossittensis*
*Leuciscus idus* (ide, säyne)
*Caryophyllaeides fennica*
*Proteocephalus torulosus*
*Leuciscus leuciscus* (common dace, seipi)
*Caryophyllaeus laticeps*
*Caryophyllaeides fennica*
*Ligula intestinalis* (l)
*Proteocephalus torulosus*
*Phoxinus phoxinus* (Eurasian minnow, mutu)
*Ligula intestinalis* (l)
*Rutilus rutilus* (common roach, särki)
*Caryophyllaeus laticeps*
*Caryophyllaeides fennica*
*Ligula intestinalis* (l)
*Proteocephalus torulosus*
*Scardinius erythrophtalmus* (common rudd, sorva)
*Caryophyllaeides fennica*
**Osmeridae (smelts, kuoreet)**
*Osmerus eperlanus* (European smelt, kuore)
*Diphyllobothrium dendriticum* (l)
*Diphyllobothrium ditremum* (l)
*Triaenophorus nodulosus* (l)
*Proteocephalus tetrastomus*
**Salmonidae (salmonids, lohet)**
*Coregonus lavaretus* (European whitefish, siika)
*Cyathocephalus truncatus*
*Diphyllobothrium dendriticum* (l)
*Diphyllobothrium ditremum* (l)
*Eubothrium crassum*
*Triaenophorus crassus* (l)
*Proteocephalus longicollis*
*Coregonus albula* (vendace, muikku)
*Diphyllobothrium dendriticum* (l)
*Diphyllobothrium ditremum* (l)
*Triaenophorus crassus* (l)
*Proteocephalus longicollis*
*Salmo salar* (Atlantic salmon, lohi)
*Diphyllobothrium dendriticum* (l)
*Eubothrium crassum*
*Triaenophorus nodulosus* (l)
*Salmo trutta* (brown trout, taimen)
*Cyathocephalus truncatus*
*Diphyllobothrium dendriticum* (l)
*Diphyllobothrium ditremum* (l)
*Eubothrium crassum*
*Eubothrium salvelini*
*Triaenophorus nodulosus* (l)
*Salvelinus alpinus* (Arctic char, nieriä)
*Diphyllobothrium dendriticum* (l)
*Diphyllobothrium ditremum* (l)
*Eubothrium salvelini*
*Triaenophorus crassus* (l)
*Proteocephalus longicollis*
*Oncorhynchus mykiss* (rainbow trout, kirjolohi)
*Triaenophorus crassus* (l)
*Thymallus thymallus* (grayling, harjus)
**Cyathocephalus truncatus*
*Triaenophorus crassus* (l)
*Proteocephalus thymalli*
**Clupeidae (clupeids, sillit)**
*Clupea harengus membras* (Baltic herring, silakka)
*Eubothrium crassum*
**Gasterosteidae (sticklebacks, piikkikalat)**
*Gasterosteus aculeatus* (three-spined stickleback, kolmipiikki)
*Diphyllobothrium dendriticum* (l)
*Diphyllobothrium ditremum* (l)
*Schistocephalus solidus* (l)
*Triaenophorus nodulosus* (l)
*Proteocephalus filicollis*
*Pungitius pungitius* (ninespine stickleback, kymmenpiikki)
*Diphyllobothrium ditremum* (l)
*Schistocephalus pungitii* (l)
*Triaenophorus nodulosus* (l)
*Proteocephalus ambiguus*
**Cottidae (cottids, simput)**
*Cottus gobio* (bullhead, kivisimppu)
*Schistocephalus cotti* (l)
*Triaenophorus nodulosus* (l)
*Myoxocephalus scorpius* (shorthorn sculpin, isosimppu)
*Bothriocephalus scorpii*
*Proteocephalus gobiorum*
*Triglopsis quadricornis* (fourhorn sculpin, härkäsimppu)
*Diphyllobothrium dendriticum* (l)
*Bothriocephalus scorpii*
*Proteocephalus gobiorum*
*Taurulus bubalis* (long-spined bullhead, piikkisimppu)
*Bothriocephalus scorpii*
**Lotidae (lings, mateet)**
*Lota lota* (burbot, made)
*Diphyllobothrium dendriticum* (l)
*Diphyllobothrium ditremum* (l)
*Diphyllobothrium latum* (l)
*Eubothrium rugosum*
*Triaenophorus nodulosus* (l)
**Gadidae (cods, turskakalat)**
*Gadus morhua* (Atlantic cod, turska)
*Diplocotyle olrikii*
**Abothrium gadi*

**AVES (birds, linnut)**
**Anseriformes (waterfowl, sorsalinnut)**
*Anas acuta* (northern pintail, jouhisorsa)
*Drepanidolepis spinulosa*
*Diorchis inflata*
*Diorchis stefanskii*
*Drepanidolepis anatina*
*Fimbriaria fasciolaris*
*Microsomacanthus abortiva*
*Microsomacanthus arcuata*
*Microsomacanthus collaris*
*Microsomacanthus paracompressa*
*Retinometra macracanthos*
*Sobolevicanthus dafilae*
*Anas clypeata* (northern shoveler, lapasorsa)
*Diorchis ransomi*
*Fimbriaria fasciolaris*
*Microsomacanthus arcuata*
*Microsomacanthus collaris*
*Microsomacanthus compressa*
*Sobolevicanthus gracilis*
*Anas crecca* (common teal, tavi)
*Drepanidolepis spinulosa*
*Aploparaksis furcigera*
*Dicranotaenia coronula*
*Diorchis elisae*
*Diorchis stefanskii*
*Diorchis ransomi*
*Drepanidolepis anatina*
*Fimbriaria fasciolaris*
*Microsomacanthus arcuata*
*Microsomacanthus collaris*
*Microsomacanthus compressa*
*Microsomacanthus paracompressa*
*Sobolevicanthus dafilae*
*Sobolevicanthus octacanthus*
*Sobolevicanthus gracilis*
*Sobolevicanthus krabbeella*
*Anas penelope* (Eurasian wigeon, haapana)
*Drepanidolepis spinulosa*
*Aploparaksis furcigera*
*Dicranotaenia coronula*
*Diorchis stefanskii*
*Diorchis asiatica*
*Diploposthe laevis*
*Drepanidolepis anatina*
*Drepanidotaenia lanceolata*
*Microsomacanthus compressa*
*Retinometra macracanthos*
*Anas platyrhynchos* (mallard, sinisorsa)
*Drepanidolepis spinulosa*
*Aploparaksis furcigera*
*Dicranotaenia coronula*
*Diorchis elisae*
*Diorchis inflata*
*Diorchis stefanskii*
*Diorchis ransomi*
*Drepanidolepis anatina*
*Fimbriaria fasciolaris*
*Microsomacanthus collaris*
*Microsomacanthus paracompressa*
*Retinometra macracanthos*
*Sobolevicanthus octacanthus*
*Sobolevicanthus gracilis*
*Anas querquedula* (garganey, heinätavi)
*Aploparaksis furcigera*
*Diorchis elisae*
*Diorchis stefanskii*
*Drepanidotaenia lanceolata*
*Fimbriaria fasciolaris*
*Sobolevicanthus octacanthus*
*Anser fabalis* (bean goose, metsähanhi)
*Wardium creplini*
*Aythya ferina* (common pochard, punasotka)
*Diploposthe laevis*
*Microsomacanthus collaris*
*Aythya fuligula* (tufted duck, tukkasotka)
*Drepanidolepis spinulosa*
*Aploparaksis furcigera*
*Dicranotaenia coronula*
*Diorchis elisae*
*Fimbriaria fasciolaris*
*Microsomacanthus arcuata*
*Microsomacanthus compressa*
*Microsomacanthus paracompressa*
*Sobolevicanthus dafilae*
*Sobolevicanthus octacanthus*
*Sobolevicanthus gracilis*
*Sobolevicanthus krabbeella*
*Aythya marila* (greater scaup, lapasotka)
*Microsomacanthus compressa*
*Retinometra macracanthos*
*Bucephala clangula* (common goldeneye, telkkä)
*Schistocephalus solidus*
*Aploparaksis furcigera*
*Dicranotaenia coronula*
*Cygnus cygnus* (whooper swan, laulujoutsen)
*Wardoides nyrocae*
*Melanitta fusca* (velvet scoter, pilkkasiipi)
*Drepanidolepis* sp. 1
*Drepanidolepis* sp. 2
*Dicranotaenia coronula*
*Mergus merganser* (common merganser, isokoskelo)
*Diphyllobothrium ditremum*
*Confluaria multistriata*?
*Fimbriaria fasciolaris*
*Ligula intestinalis*
*Tschertkovilepis tenuirostris*
*Mergus serrator* (red-breasted merganser, tukkakoskelo)
*Ligula intestinalis*
*Schistocephalus solidus*
**Fimbriaria fasciolaris*
**Sobolevicanthus gracilis*
*Somateria mollissima* (common eider, haahka)
**Fimbriaria fasciolaris*
*Microsomacanthus diorchis*
*Microsomacanthus microsoma*
*Microsomacanthus paramicrosoma*
**Galliformes (gamebirds, kanalinnut)**
*Lagopus lagopus* (willow ptarmigan, riekko)
*Paroniella urogalli*
*Skrjabinia cesticillus*
*Hymenolepis* (s.l.) sp.
*Lyrurus tetrix* (black grouse, teeri)
*Paroniella urogalli*
*Skrjabinia cesticillus*
*Hymenolepis* (s.l.) sp.
*Perdix perdix* (grey partridge, peltopyy)
*Paroniella urogalli*
*Tetrao urogallus* (western capercaillie,metso)
*Paroniella urogalli*
*Skrjabinia cesticillus*
*Hymenolepis* (s.l.) sp.
*Tetrastes bonasia* (hazel grouse, pyy)
*Paroniella urogalli*
*Skrjabinia cesticillus*
*Hymenolepis* (s.l.) sp.
**Gaviiformes (loons/divers, kuikkalinnut)**
*Gavia arctica* (black-throated loon/diver, kuikka)
*Diphyllobothrium ditremum*
*Ligula intestinalis*
*Biglandatrium biglandatrium*
*Dubininolepis rostellata*
*Gavia stellata* (red-throated loon/diver, kaakkuri)
*Dubininolepis rostellata*
*Neovalipora parvispine*
*Tethrabothrius macrocephalus*
*Tetrabothrius mawsoni*
**Podicipediformes (grebes, uikkulinnut)**
*Podiceps cristatus* (great crested grebe, silkkiuikku)
*Ligula intestinalis*
*Tetrabothrius macrocephalus*
*Tetrabothrius mawsoni*
*Confluaria pseudofurcifera*
*Podiceps grisegena* (red-necked grebe, härkälintu)
*Confluaria furcifera*
**Pelecaniformes (pelicans, cormorants etc., pelikaanilinnut)**
*Phalacrocorax carbo* (great cormorant, merimetso)
*Ligula intestinalis*
**Accipitriformes (hawks and eagles, päiväpetolinnut)**
*Buteo buteo* (common buzzard, hiirihaukka)
*Cladotaenia globifera*
*Buteo lagopus* (rough-legged buzzard, piekana)
*Cladotaenia globifera*
**Charadriiformes (shorebirds, rantalinnut)**
*Actitis hypoleucos* (common sandpiper, rantasipi)
**Anomotaenia arionis*
**Kowalewskiella cingulifera*
*Arenaria interpres* (ruddy turnstone, karikukko)
*Schistocephalus solidus*
*Calidris alpina* (dunlin, suosirri)
**Sacciuterina paradoxa*
*Aploparaksis crassirostris*
*Cepphus grylle* (black guillemot, riskilä)
**Alcataenia campylacantha*
*Tethrabothrius macrocephalus*
*Charadrius hiaticula* (common ringed plover, tylli)
**Anomoatenia microrhyncha*
*Larus argentatus* (European herring gull, harmaalokki)
*Diphyllobothrium ditremum*
*Ligula intestinalis*
*Tetrabothrius mawsoni*
*Larus canus* (common gull, kalalokki)
*Alcataenia larina*
*Larus fuscus* (lesser black-backed gull, selkälokki)
*Ligula intestinalis*
*Limicola falcinella* (broad-billed sandpiper, jänkäsirriäinen)
*Aploparaksis crassirostris*
*Limosa lapponica* (bar-tailed godwit, punakuiri)
*Ophryocotyle proteus*
*Numenius arquata* (Eurasian curlew, kuovi)
*Aploparaksis filum* s.l.
*Dictymetra laevigata*
*Phalaropus lobatus* (red-necked phalarope, vesipääsky)
**Dictymetra laevigata*
*Philomachus pugnax* (ruff, suokukko)
**Anomoatenia microrhyncha*
*Pluvialis apricaria* (European golden plover, kapustarinta)
*Nototaenia brevis*
*Riparia riparia* (sand martin, törmäpääsky)
*Angularella* sp.
*Scolopax rusticola* (Eurasian woodcock, lehtokurppa)
*Anomotaenia globulus*
*Aploparaksis filum* s.l.
*Fuhrmannolepis* sp.
*Sacciuterina paradoxa*
*Tringa glareola* (wood sandpiper, liro)
*Trichocephaloidis* sp.
*Aploparaksis crassirostris*
*Aploparaksis filum* s.l.
*Sterna hirundo* (common tern, kalatiira)
*Schistocephalus solidus*
*Sterna paradisaea* (Arctic tern, lapintiira)
*Schistocephalus solidus*
*Uria aalge* (common murre/guillemot, etelänkiisla)
*Tethrabothrius macrocephalus*
**Columbiformes (pigeons and doves, kyyhkylinnut)**
*Columba palumbus* (common wood pigeon, sepelkyyhky)
*Dilepis undula*
**Strigiformes (owls, pöllölinnut)**
*Strix uralensis* (Ural owl, viirupöllö)
*Paruterina candelabraria*
*Aegolius funereus* (Tengmalm’s owl, helmipöllö)
*Paruterina candelabraria*
**Apodiformes (swifts and hummingbirds, kirskulinnut)**
*Apus apus* (common swift, tervapääsky)
*Neoliga depressa*
*Notopentorchis cyathiformis*
**Piciformes (woodpeckers, tikkalinnut)**
*Dendrocopos leucotos* (white-backed woodpecker, valkoselkätikka)
*Liga crateriformis*
*Dendrocopos major* (great spotted woodpecker, käpytikka)
*Liga crateriformis*
*Dryocopus martius* (black woodpecker, palokärki)
*Railletina frontina*
*Picoides tridactylus* (Eurasian three-toed woodpecker, pohjantikka)
*Orthoskrjabinia* sp.
*Picus canus* (grey-headed woodpecker, harmaapäätikka)
*Liga crateriformis*
**Passeriformes (passerines, varpuslinnut)**
*Anthus trivialis* (tree pipit, metsäkirvinen)
*Anonchotaenia globata*
*Corvus corone* (carrion crow, varis)
*Dilepis undula*
*Spiniglans constricta*
*Passerilepis crenata*
*Delichon urbica* (common house martin, räystäspääsky)
**Hirundinicola parvirostris*
*Fringilla montifringilla* (brambling, järripeippo)
*Monosertum parinum*
*Hirundo rustica* (barn swallow, haarapääsky)
*Hirundinicola parvirostris*
*Lanius collurio* (red-backed shrike, pikkulepinkäinen)
*Biuterina* sp.
*Paruterina parallelepipeda*
*Motacilla alba* (white wagtail, västäräkki)
**Sobolevitaenia borealis*
*Parus major* (great tit, talitiainen)
*Passerilepis parina*
*Pica pica* (magpie, harakka)
*Dilepis undula*
*Passerilepis stylosa*
*Spiniglans sharpiloi*
*Sturnus vulgaris* (common starling, kottarainen)
*Wardium farciminosa*
*Turdus iliacus* (redwing, punakylkirastas)
*Dilepis undula*
*Passerilepis crenata*
*Turdus philomelos* (song thrush, laulurastas)
*Dilepis undula*
*Turdus pilaris* (fieldfare, räkättirastas)
*Dilepis undula*
*Passerilepis crenata*
*Turdus viscivorus* (mistle thrush, kulorastas)
*Dilepis undula*
*Passerilepis crenata*
**MAMMALIA (mammals, nisäkkäät)**
**Soricidae (shrews, päästäiset)**
*Sorex araneus* (common/Eurasian shrew, metsäpäästäinen)
*Dilepis undula*
*Hepatocestus hepaticus*
*Monocercus arionis*
*Ditestolepis diaphana*
*Gulyaevilepis tripartita*
*Lineolepis scutigera*
*Neoskrjabinolepis merkushevae*
*Neoskrjabinolepis schaldybini*
*Neoskrjabinolepis singularis*
*Pseudobotrialepis globosoides*
*Soricinia infirma*
*Spasskylepis ovaluteri*
*Staphylocystis furcata*
*Staphylocystoides stefanskii*
*Urocystis prolifer*
*Vigisolepis spinulosa*
*Mesocestoides lineatus* (l)
*Sorex caecutiens* (Laxmann’s shrew, idänpäästäinen)
*Monocercus arionis*
*Ditestolepis diaphana*
*Gulyaevilepis tripartita*
*Lineolepis scutigera*
*Neoskrjabinolepis merkushevae*
*Neoskrjabinolepis schaldybini*
*Neoskrjabinolepis singularis*
*Pseudobotrialepis globosoides*
*Soricinia infirma*
*Spasskylepis ovaluteri*
*Staphylocystis furcata*
*Vigisolepis spinulosa*
*Sorex minutus* (Eurasian pygmy shrew, vaivaispäästäinen)
*Monocercus arionis*
*Ditestolepis diaphana*
*Neoskrjabinolepis schaldybini*
*Pseudobotrialepis globosoides*
*Staphylocystoides stefanskii*
*Vigisolepis spinulosa*
*Sorex isodon* (taiga shrew, mustapäästäinen)
*Monocercus arionis*
*Ditestolepis diaphana*
*Ditestolepis* sp.
*Neoskrjabinolepis schaldybini*
*Vigisolepis spinulosa*
*Neomys fodiens* (Eurasian water shrew, vesipäästäinen)
*Polycercus* sp.
*Vigisolepis spinulosa*
**Vespertilionidae (vesper bats, siipat)**
*Eptesicus nilssoni* (northern bat, pohjanlepakko)
*Vampirolepis* sp.
**Leporidae (rabbits and hares, jänikset)**
*Lepus europaeus* (European hare, rusakko)
*Mosgovoyia pectinata*
*Taenia pisiformis* (l)
*Lepus timidus* (mountain hare, metsäjänis)
*Mosgovoyia pectinata*
*Taenia pisiformis* (l)
*Oryctolagus cuniculus* (European rabbit, kani)
*Neoctenotaenia ctenoides*
**Muridae (Old World rats and mice, rottaeläimet)**
*Apodemus flavicollis* (yellow-necked mouse, metsähiiri)
Hymenolepis cf. diminuta
*Rodentolepis fraterna*
*Skrjabinotaenia lobata*
*Mesocestoides lineatus* (l)
*Hydatigera taeniaeformis* s.l. (l)
*Micromys minutus* (harvest mouse, vaivaishiiri)
*Nomadolepis* sp.
*Mus musculus* (house mouse, kotihiiri)
*Catenotaenia pusilla*
*Rattus norvegicus* (brown rat, isorotta)
*Hydatigera taeniaeformis* s.l. (l)
**Cricetidae (cricetids, hamsterit ja myyrät)**
*Arvicola amphibius* (European water vole, vesimyyrä)
Anoplocephaloides cf. dentata
*Eurotaenia gracilis*
*Paranoplocephala omphalodes*
*Lemmus lemmus* (Norwegian lemming, tunturisopuli)
Anoplocephaloides cf. dentata
*Eurotaenia gracilis*
*Lemminia fellmani*
*Microtus agrestis* (field vole, peltomyyrä)
Anoplocephaloides cf. dentata
*Eurotaenia gracilis*
Microcephaloides cf. variabilis
*Microticola blanchardi*
*Paranoplocephala omphalodes*
Hymenolepis (s.l.) asymmetrica
*Mesocestoides lineatus* (l)
*Hydatigera taeniaeformis* s.l. (l)
*Versteria mustelae* (l)
*Microtus levis* (East European vole, idänkenttämyyrä)
*Paranoplocephala omphalodes*
*Taenia polyacantha* (l)
*Microtus oeconomus* (root vole/tundra vole, lapinmyyrä)
Anoplocephaloides cf. dentata
*Eurotaenia gracilis*
Microcephaloides cf. variabilis
*Microticola blanchardi*
*Paranoplocephala jarrelli*
*Taenia polyacantha* (l)
*Versteria mustelae* (l)
*Myodes glareolus* (bank vole, metsämyyrä)
*Eurotaenia gracilis*
*Paranoplocephala omphalodes*
*Paranoplocephala kalelai*
*Catenotaenia henttoneni*
*Cladotaenia globifera* (l)
*Mesocestoides lineatus* (l)
*Taenia martis* (l)
*Taenia polyacantha* (l)
*Hydatigera taeniaeformis* s.l. (l)
*Versteria mustelae* (l)
*Myodes rufocanus* (grey-sided vole, harmaakuvemyyrä)
Anoplocephaloides cf. dentata
*Eurotaenia gracilis*
Microcephaloides cf. variabilis
*Paranoplocephala kalelai*
*Mesocestoides lineatus* (l)
*Versteria mustelae* (l)
*Myodes rutilus* (red vole/northern red-backed vole, punamyyrä)
*Eurotaenia gracilis*
*Paranoplocephala kalelai*
*Catenotaenia henttoneni*
*Mesocestoides lineatus* (l)
*Taenia martis* (l)
*Taenia polyacantha* (l)
*Hydatigera taeniaeformis* s.l. (l)
*Versteria mustelae* (l)
*Ondatra zibethicus* (muskrat, piisami)
*Hydatigera taeniaeformis* s.l. (l)
**Sciuridae (squirrels, oravat)**
*Sciurus vulgaris* (Eurasian red squirrel, orava)
*Catenotaenia dendritica*
**Felidae (cats, kissaeläimet)**
*Felis catus* (domestic cat, kissa)
*Hydatigera taeniaeformis* s.l.
*Lynx lynx* (Eurasian lynx, ilves)
*Spirometra* sp.
*Taenia laticollis*
*Taenia* sp.
*Hydatigera taeniaeformis* s.l.
**Mustelidae (mustelids, näätäeläimet)**
*Lutra lutra* (otter, saukko)
*Versteria mustelae* (l)
*Martes martes* (European pine marten, näätä)
*Mesocestoides lineatus*
*Meles meles* (European badger, mäyrä)
*Atriotaenia incisa*
*Mesocestoides lineatus*
**Canidae (canids, koiraeläimet)**
*Canis lupus* (wolf, susi)
*Mesocestoides lineatus*
*Taenia hydatigena*
*Taenia krabbei*
*Echinococcus canadensis*
*Canis lupus familiaris* (dog, koira)
*Diphyllobothrium latum*
*Dipylidium caninum*
*Taenia pisiformis*
*Nyctereutes procyonoides* (raccoon dog, supikoira)
*Polycercus* sp.
*Vulpes vulpes* (red fox, kettu)
*Diphyllobothrium latum*
*Mesocestoides litteratus*
*Taenia polyacantha*
**Ursidae (bears, karhut)**
*Ursus arctos* (brown bear, karhu)
*Taenia arctos*
**Phocidae (true seals, hylkeet)**
*Pusa hispida saimensis* (Saimaa ringed seal, saimaannorppa)
*Diphyllobothrium ditremum*
*Triaenophorus nodulosus*
*Pusa hispida botnica* (Baltic ringed seal, itämerennorppa)
*Schistocephalus solidus*
**Equidae (horses, hevoset)**
*Equus caballus* (horse, hevonen)
*Anoplocephala perfoliata*
*Echinococcus equinus* (l)
**Cervidae (deer, hirvieläimet)**
*Alces alces* (Eurasian elk/moose, hirvi)
*Moniezia expansa*
*Taenia arctos* (l)
*Taenia hydatigena* (l)
*Taenia* sp. (l)
*Echinococcus canadensis* (l)
*Capreolus capreolus* (European roe deer, metsäkauris)
*Taenia* sp. (l)
*Rangifer tarandus* (reindeer, poro/peura)
Moniezia cf. benedeni
*Taenia krabbei* (l)
*Echinococcus canadensis* (l)
**Bovidae (cloven-hoofed mammals, onttosarviset)**
*Ovis aries* (sheep, lammas)
**Moniezia expansa*
*Taenia hydatigena* (l)
*Bos taurus* (cow/cattle, lehmä/nauta)
*Moniezia benedeni*
*Taenia saginata* (l)
**Suidae (pigs, siat)**
*Sus scrofa* (domestic pig, sika)
*Taenia hydatigena* (l)
**Hominidae (great apes, isot ihmisapinat)**
*Homo sapiens* (man, ihminen)
*Diphyllobothrium latum*
*Taenia saginata*
*Taenia solium*/*Taenia solium* (l)
*Echinococcus granulosus* (l)
*Echinococcus multilocularis* (l)
